# COX5B Regulates MAVS-mediated Antiviral Signaling through Interaction with ATG5 and Repressing ROS Production

**DOI:** 10.1371/journal.ppat.1003086

**Published:** 2012-12-20

**Authors:** Yuanyuan Zhao, Xiaofeng Sun, Xuanli Nie, Liwei Sun, Tie-shan Tang, Dahua Chen, Qinmiao Sun

**Affiliations:** 1 State Key Laboratory of Biomembrane and Membrane Biotechnology, Institute of Zoology, Chinese Academy of Sciences, Chaoyang District, Beijing, P. R. China; 2 State Key Laboratory of Reproductive Biology, Institute of Zoology, Chinese Academy of Sciences, Chaoyang District, Beijing, P. R. China; Yale University School of Medicine, United States of America

## Abstract

Innate antiviral immunity is the first line of the host defense system that rapidly detects invading viruses. Mitochondria function as platforms for innate antiviral signal transduction in mammals through the adaptor protein, MAVS. Excessive activation of MAVS-mediated antiviral signaling leads to dysfunction of mitochondria and cell apoptosis that likely causes the pathogenesis of autoimmunity. However, the mechanism of how MAVS is regulated at mitochondria remains unknown. Here we show that the Cytochrome c Oxidase (CcO) complex subunit COX5B physically interacts with MAVS and negatively regulates the MAVS-mediated antiviral pathway. Mechanistically, we find that while activation of MAVS leads to increased ROS production and COX5B expression, COX5B down-regulated MAVS signaling by repressing ROS production. Importantly, our study reveals that COX5B coordinates with the autophagy pathway to control MAVS aggregation, thereby balancing the antiviral signaling activity. Thus, our study provides novel insights into the link between mitochondrial electron transport system and the autophagy pathway in regulating innate antiviral immunity.

## Author Summary

Pattern recognition receptors are vital to innate immunity. In the antiviral innate immunity, retinoic acid-inducible gene-I (RIG-I)-like receptors (RLRs), such as RIG-I and MDA5, sense viral RNAs through their C-terminal helicase domains, then initiate the antiviral response through interaction with the essential adaptor protein MAVS, which is located in mitochondrial outer membrane. Although cumulative studies have showed that mitochondria-associated MAVS plays an important role in antiviral signaling, much remains unknown about the mechanism of MAVS activity related to mitochondrial membrane localization. In this article we demonstrate that the CcO complex subunit COX5B negatively regulates the MAVS-mediated antiviral pathway through interaction with MAVS. At the mechanistic level, we show that COX5B inhibits MAVS-mediated antiviral pathway by suppressing ROS production, and coordinating with the autophagy pathway to control MAVS aggregation. Our data support a notion that mitochondrial electron transport system coordinates with the autophagy pathway to regulate MAVS-mediated signaling for a tight control of innate antiviral immunity.

## Introduction

Innate immunity is the first line of the host defense system that rapidly detects and eliminates invading microorganisms, such as bacteria, fungi and viruses. In the host cells, detection of conserved microbial molecules (pathogen-associated molecular patterns) involves several pattern recognition receptors (PRRs), including membrane bound Toll-like receptors (TLRs) and cytosolic receptors, such as retinoic acid-inducible gene-I (RIG-I)-like receptors (RLRs) [1], [2].

Detection of viral infection by viral-sensing receptors, TLRs and RLRs, triggers signaling cascades that lead to production of type I interferons (IFNs), as well as subsequent innate antiviral responses that suppress virus replication [3]–[5]. Unlike the Toll-like receptor-mediated antiviral response, RLRs receptors RIG-I and MDA5 function as cytoplasmic sensors for viral RNA recognition [2]. Both RIG-I and MDA5 contain a C-terminal DExD/H-box RNA helicase domain that directly senses viral RNAs and two caspase recruitment domains (CARDs) at their N terminus that mediate downstream signaling through interaction with the essential adaptor protein MAVS (also known as IPS-1, VISA, or CARDIF) [6]–[9]. MAVS then further recruits the TBK1 and IKK complex to activate transcription factors IRF3/IRF7 and NF-κB respectively, which coordinately induce type I IFNs production and elicit the innate response [6], [10].

MAVS contains a C-terminal transmembrane domain (TM) that targets it to the mitochondrial outer membrane. Deletion TM or replacement of this TM with other membrane locations leads to loss of function of MAVS, indicating the essential role of the mitochondrial localization of MAVS in the antiviral signaling pathway [6]. However, the mechanism of MAVS activity related to mitochondrial membrane localization remains poorly understood. Recent studies have identified several mitochondrial membrane-associated proteins such as NLRX1 [11], [12], RNF5 [13], MFN1 [14], MFN2 [15] that either negatively or positively regulate MAVS activity, indicating that the mitochondrial membrane at least provides a platform for the MAVS-mediated antiviral signal transduction. Interestingly, recent studies also revealed that overexpression of MAVS leads to dysfunction of mitochondria, as well as cell apoptosis [16], that potentially causes the pathogenesis of autoimmunity and infectious diseases, suggesting that excessive activation of the antiviral signaling pathway could be a damage signal to infected cells. Thus, MAVS signaling and function must be under quality control to maintain immune balance.

Reactive oxygen species (ROS) and autophagy have emerged as important players in regulating innate immunity, particularly in the antiviral signaling pathway [17]–[19]. Previous studies have revealed that the autophagy pathway controls RIG-I/MAVS signaling by repressing ROS production [18], [20]. However, the molecular basis of how ROS and autophagy regulate RIG-I/MAVS signaling is unknown. In this study, we show that the CcO complex subunit COX5B physically interacts with MAVS and negatively regulates the MAVS-mediated antiviral pathway, revealing a novel function of COX5B, in addition to its role in the transfer of electrons in mitochondria. Importantly, our study suggests that COX5B coordinates with the autophagy pathway to repress mitochondrial ROS production and control MAVS aggregation, thereby balancing the antiviral signaling activity.

## Results

### Identification of COX5B as a MAVS-interacting partner

**Figure 1:**
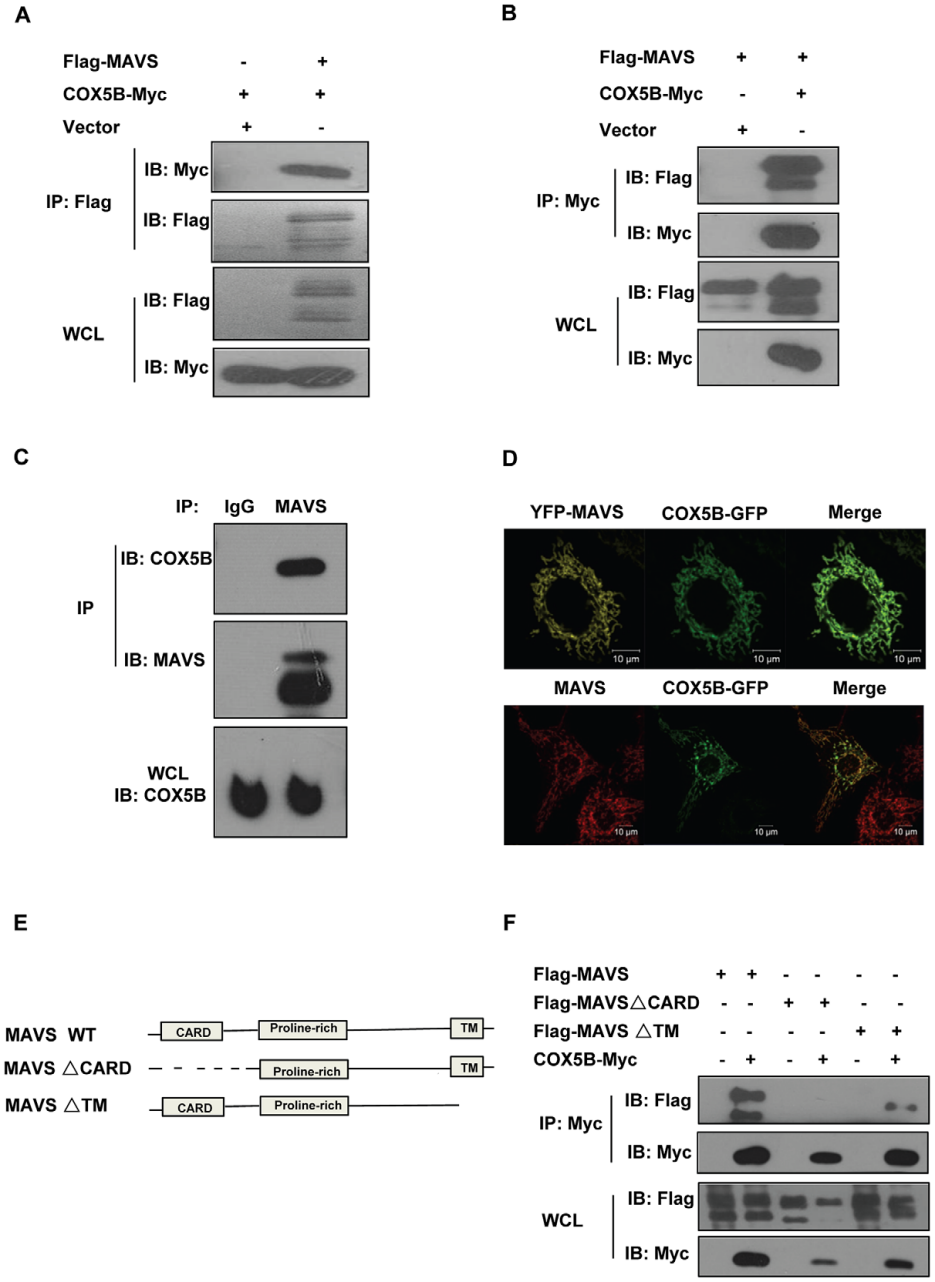
COX5B interacts with MAVS. (A and B) HEK293 cells were transfected with combinations of DNA constructs as indicated. Twenty-four hours after transfection, cell lysates were prepared, immunoprecipitated with anti-Flag beads (A) or with anti-Myc antibody (B), followed by immunoblot analysis with the indicated antibodies. WCL (bottom), expression of transfected proteins in whole-cell lysates. (C) HEK293 cell lysates were prepared, immunoprecipitated with anti-MAVS antibody or control IgG, followed by immunoblot analysis. (D) Hela cells were transfected with COX5B-GFP and control vector or YFP-MAVS for 36 h. Cells were fixed, then stained with anti-MAVS antibody (Bottom) or mounted onto slides directly (Top), and imaged by confocal microscopy. (E) Schematic diagram of MAVS and truncated mutants. (F) HEK293 cells were transfected with the indicated plasmids, cell lysates were immunoprecipitated with anti-Flag beads, followed by immunoblot analysis.

To better understand the molecular mechanism underlying the action of MAVS in the antiviral signaling pathway, we carried out a yeast two-hybrid screen to search for MAVS-interacting proteins using the fragment containing CARD domain of MAVS as bait (Table S1 in Text S1). Sequence analysis revealed that one of the positive clones encodes the full length subunit of the cytochrome c oxidase complex (CcO), COX5B [21]. To confirm this, we then performed co-immunoprecipitation experiments to determine whether COX5B interacts with MAVS in human embryonic kidney (HEK293) cells. As shown in Figures 1A, 1B and Figure S1A in Text S1, the epitope-tagged COX5B but not COX5A, another component of CcO complex, could co-immunoprecipitate with MAVS in transfected HEK293 cells. Consistent with this observation, we found that endogenous COX5B and MAVS proteins also interact with each other (Figure 1C). In agreement with this, immunostaining analysis revealed that a significant portion of COX5B was co-localized with MAVS at the mitochondria when cells expressed GFP-tagged COX5B and YFP-tagged MAVS (Figure 1D, top panel). Additionally, significant overlap was also observed between the endogenous MAVS and GFP-tagged COX5B (Figure 1D, bottom panel). Thus, our findings support the concept that COX5B physically interacts with MAVS, and their interaction likely occurs at the mitochondria in cultured cells.

Consistent with the yeast two-hybrid assay, our domain-mapping experiments revealed that MAVS interacts with COX5B through its CARD domain, since the mutant form of MAVS, MAVSΔCARD, which lacks the CARD domain, failed to interact with COX5B (Figures 1E–1F). Prior studies have shown that the CARD domain in MAVS is essential for the MAVS-mediated antiviral signaling [6]. Thus, our data raises a possibility that COX5B might have a role in regulating antiviral signaling through its interaction with MAVS.

### Overexpression of COX5B attenuates the MAVS-mediated antiviral signaling

**Figure 2:**
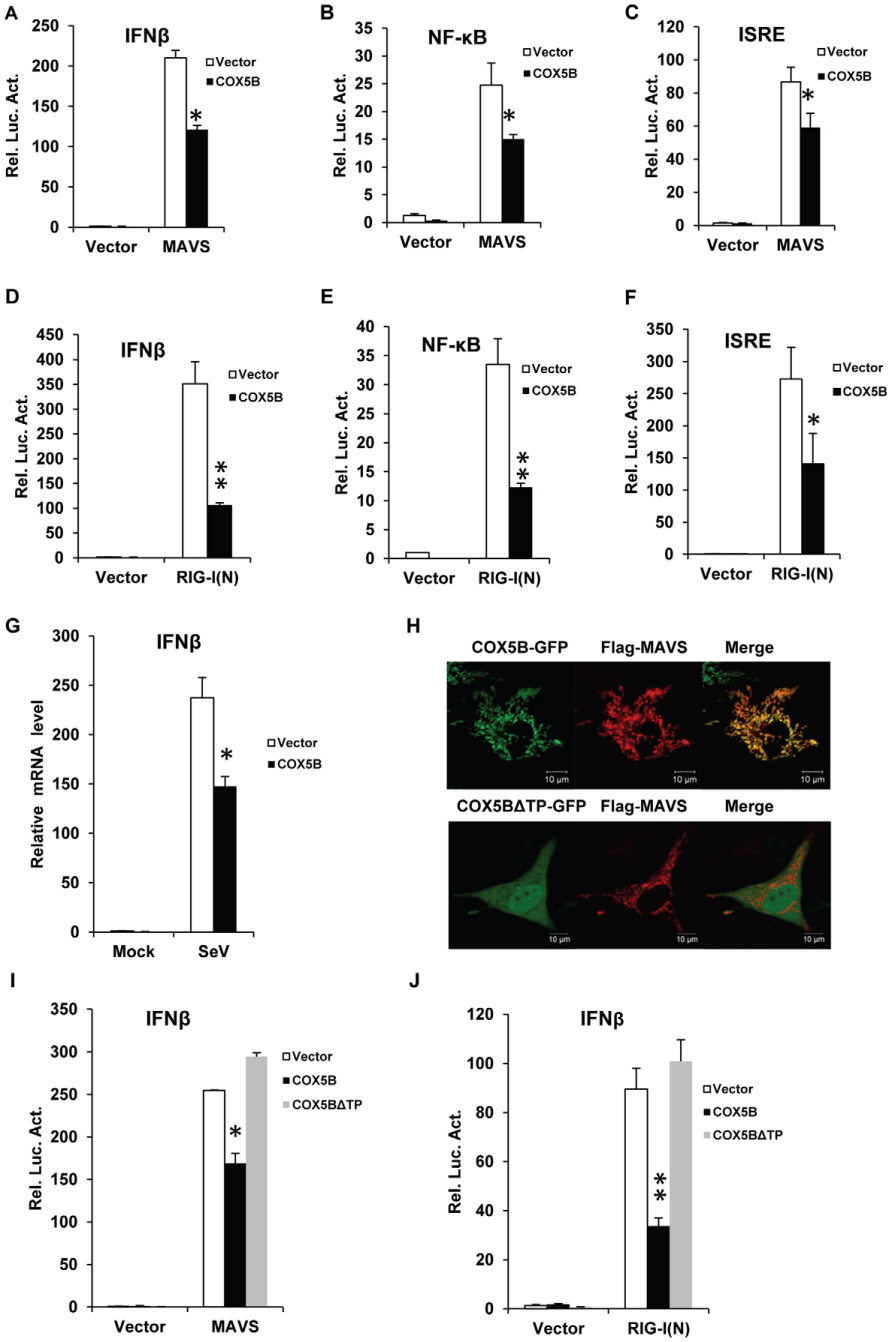
Overexpression of COX5B suppresses MAVS-mediated antiviral signaling. (A–C) MAVS and COX5B, or empty expression vectors were transfected in HEK293 cells together with luciferase reporter constructs driven by promoters of genes encoding IFNβ (A), NF-κB (B) or ISRE(C), as well as pRSV/LacZ as an internal control. Twenty-four hours after transfection, the luciferase activity was measured and normalized based on β-galactosidase activity. Results are presented relative to the luciferase activity in control cells. (D–F) RIG-I(N) and COX5B or empty expression vectors were transfected in HEK293 cells together with IFNβ-luc (D), NF-κB-luc (E) or IRSE-luc (F) along with pRSV/LacZ. Subsequently, cells were lysed for luciferase assays. (G) HEK293 cells were transfected with empty expression vector or COX5B for 24 h. The cells were then untreated or infected with Sendai virus (50HA units/ml) for 12 h, total RNA was isolated to check the expression of IFNβ mRNA by real-time PCR. (H) Hela cells were transfected with COX5B-GFP or COX5BΔTP-GFP and Flag-MAVS, the cells were then stained with the anti-Flag antibody and imaged by confocal microscopy. (I) HEK293 cells were transfected with the indicated constructs together with IFNβ reporter plasmids. Cells were lysed for luciferase assays after 24 h transfection. (J) HEK293 cells were transfected similarly as in (I), except that RIG-I(N) construct was used instead of MAVS. Data from A–G, I and J are representative of at least three independent experiments (mean and s.d. of duplicate or triplicate assays). *, P<0.05; **, P<0.01 versus the control groups.

Previous studies have demonstrated that overexpression of MAVS induces a robust activation of antiviral cascades, leading to expression of interferons [6]–[9]. To determine the role of COX5B in MAVS-mediated antiviral signaling, we first examined whether overexpression of COX5B affects the MAVS-induced activation of IFN-β, NF-κB, and interferon-stimulated response element (ISRE) promoters. As shown in the luciferase reporter assays (Figures 2A–2C), overexpression of MAVS in HEK293 cells was sufficient to activate the promoters for IFN-β, NF-κB and ISRE, whereas the activation of these promoters was suppressed by co-expression of COX5B compared with control. Given that overexpression of the tandem N-terminal CARD-like domains of RIG-I (designated as RIG-I(N)) also induces antiviral signaling through MAVS, we then examined whether overexpression of COX5B inhibits the function of RIG-I. As shown in Figures 2D–2F, co-expression of COX5B attenuated the activation of IFN-β, NF-κB, and ISRE promoters induced by overexpression of RIG-I(N) in HEK293 cells. We also found overexpression of COX5B reduced the levels of IFN-β mRNA induced by Sendai virus infection (Figure 2G). These data together indicate the potential negative role of COX5B in the MAVS-mediated activation of cellular signaling.

To explore the specificity of the inhibitory role of COX5B in MAVS signaling pathway, we first test whether overexpression of COX5A suppresses MAVS signaling, and found no apparent inhibition of overexpression COX5A on activation of the IFNβ promoter induced by RIG-I(N) (Figure S1B in Text S1). We next probed whether overexpression COX5B suppresses the activation of IFN-β and NF-κB promoters specifically through MAVS signaling, and found that overexpression of COX5B failed to suppress IFN-β or NF-κB activation induced by either overexpression of Trif or the treatment of TNFα (Figures S2A–S2B in Text S1). Thus, the inhibition MAVS signaling by COX5B appears to be specific.

Given that COX5B and MAVS co-localizes at the mitochondria, we then sought to test whether mitochondrial-localization is important for COX5B in anti-virus signaling pathway. A previous study proposed that a 31-residue transit peptide (TP) in the N-terminal of COX5B is required for its appropriate mitochondrial localization [22]. To determine the function of TP of COX5B in regulating antiviral signaling, we generated a mutant construct of COX5B, COX5BΔTP, which lacks TP (Figure 2H). In agreement with previous work, COX5B lacking TP was no longer overlapped with MAVS. As shown in Figure 2I, overexpression of COX5BΔTP failed to suppress the MAVS-induced activation of IFN-β promoters. Similar findings were observed when cells were co-overexpressing RIG-I(N) with COX5BΔTP (Figure 2J). These data demonstrate that COX5B negatively regulates MAVS signaling in a manner that depends on its mitochondrial localization.

### Knockdown of COX5B enhances MAVS-mediated antiviral signaling

**Figure 3:**
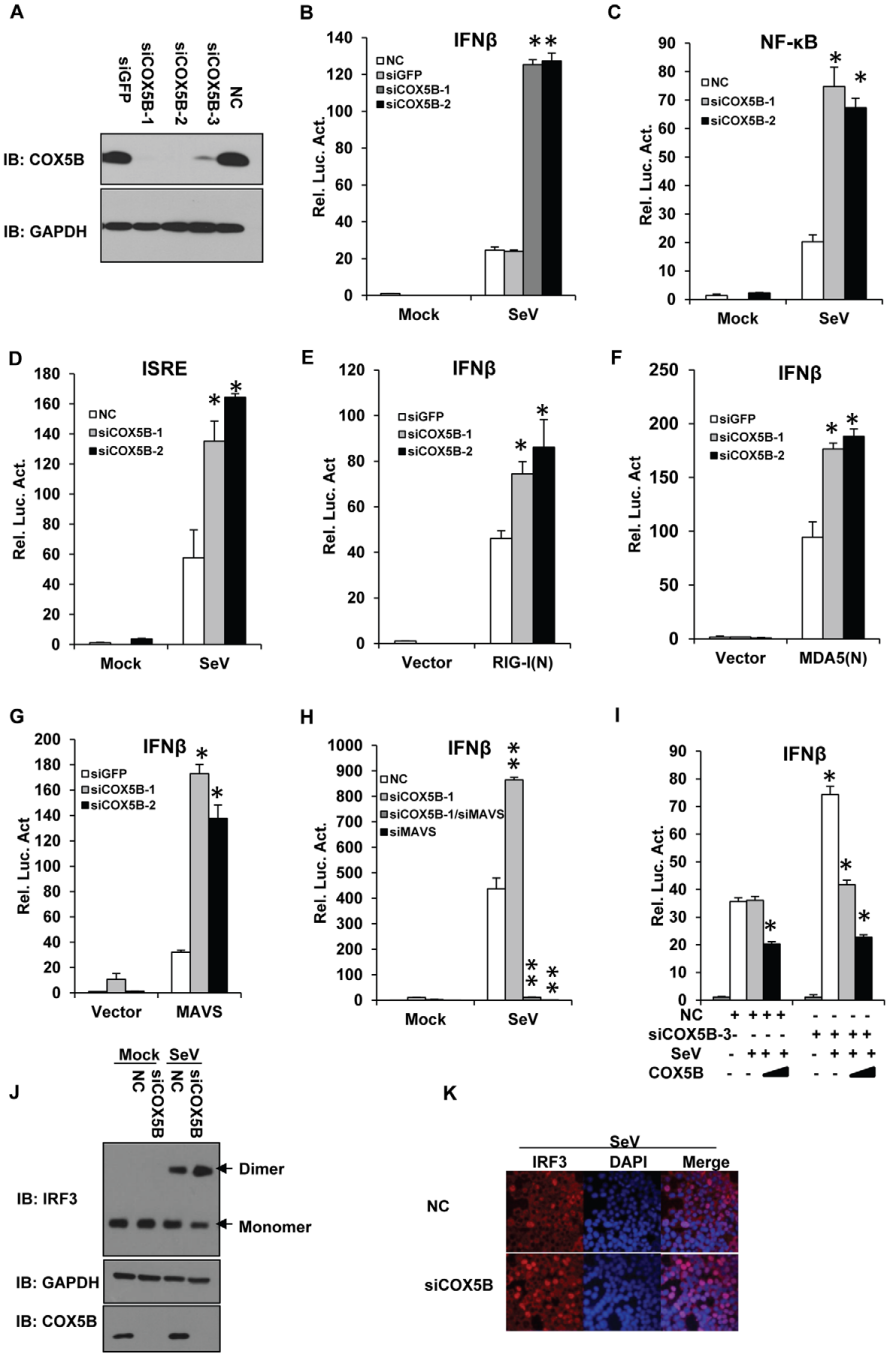
COX5B negatively regulates MAVS-mediated antiviral signaling. (A) Oligos targeting three different regions of COX5B, NC (control) or siGFP (control) were transfected into HEK293 cells, 48 h after transfection, cell lysates were analyzed by immunoblotting with the indicated antibodies. (B–D) HEK293 cells were first transfected with NC, GFP or COX5B RNAi oligos (either NC or GFP RNAi as negative control). After 48 h transfection, IFNβ (B), NF-κB (C), or ISRE (D) reporter plasmids were transfected into the RNAi cells respectively, followed by the Sendai virus (50HA unit/ml) infection for 16 h, and subsequently cells were lysed for luciferase assays. (E–G) HEK293 cells were transfected with GFP or COX5B RNAi oligos. After 48 h transfection, and the indicated expression vectors RIG-I(N) (E), MDA5(N) (F), or MAVS (G) along with reporter plasmids were transfected into the RNAi cells, followed by luciferase assay after the second transfection for 24 h. (H) HEK293 cells were transfected with 20 nM oligos as indicated, NC oligos were used to balance the equal amount of RNAi oligos, second transfection and virus infection were performed as in (B). (I) HEK293 cells were first transfected with COX5B 3′UTR RNAi oligo, 12 h after transfection, reporter plasmids and the increasing amounts of COX5B expression vectors were then transfected into the RNAi cells, 24 h after the second transfection, the cells were then infected with Sendai virus (50HA unit/ml) for 16 h. Finally, cells were lysed for luciferase assays. (J–K) Knockdown of COX5B enhances dimerization and nuclear translocation of IRF3 induced by Sendai virus infection. (J) HEK293 cells were transfected with COX5B RNAi or control oligos. Forty-eight hours after transfection, cells were infected with SeV or left uninfected for 10 h. Cell lysates were resolved by native gel electrophoresis (upper panel) or SDS-PAGE (lower panels) and analyzed with the indicated antibodies. (K) HEK293 cells were transfected with COX5B RNAi oligos as in (J), subsequently cells were infected with SeV for 9 h, stained with an IRF3 antibody and DAPI, then imaged by confocal microscopy. Data from B–I are representative of at least three independent experiments (mean and s.d. of duplicate assays). *, P<0.05; **, P<0.01 versus the control groups.

To further confirm the role of COX5B in the antiviral pathway, we then investigated the function of endogenous COX5B in the MAVS-mediated pathway. We synthesized three different pairs of siRNA oligos targeting distinct regions of COX5B, siCOX5B-1, siCOX5B-2 and siCOX5B-3, for knockdown of COX5B in cultured cells. Among them, the siCOX5B-1 and siCOX5B-2 were designed to target the open reading frame of COX5B, while the siCOX5B-3 was targeted to an untranslated region in COX5B. As shown in Figure 3A, all three RNAi oligos efficiently down-regulated the expression of endogenous COX5B in transfected HEK293 cells. Consistently, we also found that levels of ATP were reduced in COX5B knockdown cells (Figure S3A in Text S1), supporting the known function of COX5B in the ATP production. In agreement with the notion of the inhibitory role of COX5B in the MAVS-mediated pathway, we found that knockdown of COX5B significantly enhanced the activation of IFN-β, NF-κB and ISRE promoters induced by Sendai virus infection in HEK293 cells (Figures 3B–3D). Similarly, RNAi of COX5B significantly increased IFN-β promoter activity in cells with overexpression of either RIG-I(N), the N-terminal fragment of MDA5 (MDA5(N)), or MAVS (Figures 3E–3G), as well as with infection by vesicular stomatitis mutant virus (VSVΔM51), which carries a single amino acid deletion (methionine 51) in the matrix (M) protein [23]–[24] (Figure S4A in Text S1). In addition, we found that knockdown of COX5B enhanced SeV-induced activation of the IFN-β promoter depended on MAVS expression, since the activation of the IFN-β is almost completely lost in MAVS knockdown cells (Figure 3H). We noted that knockdown of COX5B enhanced SeV-induced activation of the IFN-β promoter in other cell lines, such as HepG2 cells, suggesting that the regulatory role of COX5B in RIG-I/MAVS-mediated antiviral signaling is not cell-type specific (Figure S4B in Text S1). To further validate the specificity of COX5B in antiviral signaling, we performed rescue experiments. As shown in Figure 3I, we knocked down COX5B by siRNA COX5B-3, which targets to the 3′ untranslated region in COX5B, and then reintroduced COX5B-Myc-expressing plasmids into HEK293 cells. We found that restored expression of COX5B almost fully rescued the effects of RNAi of COX5B on antiviral signaling transduction.

Given that dimerization and nuclear translocation of IRF3 are important features of activation of the RLRs pathway [25], we then tested whether knockdown of COX5B has an effect on dimerization and nuclear translocation of IRF3. As expected, we found that inactivation of COX5B by siRNA knockdown increased the dimerization, and nuclear translocation of IRF3 induced by Sendai virus infection (Figures 3J–3K). Taken together, our findings strongly indicate that COX5B acts as a negative regulator in balancing the MAVS-mediated cellular signaling.

### COX5B negatively controls the virus amplification

**Figure 4:**
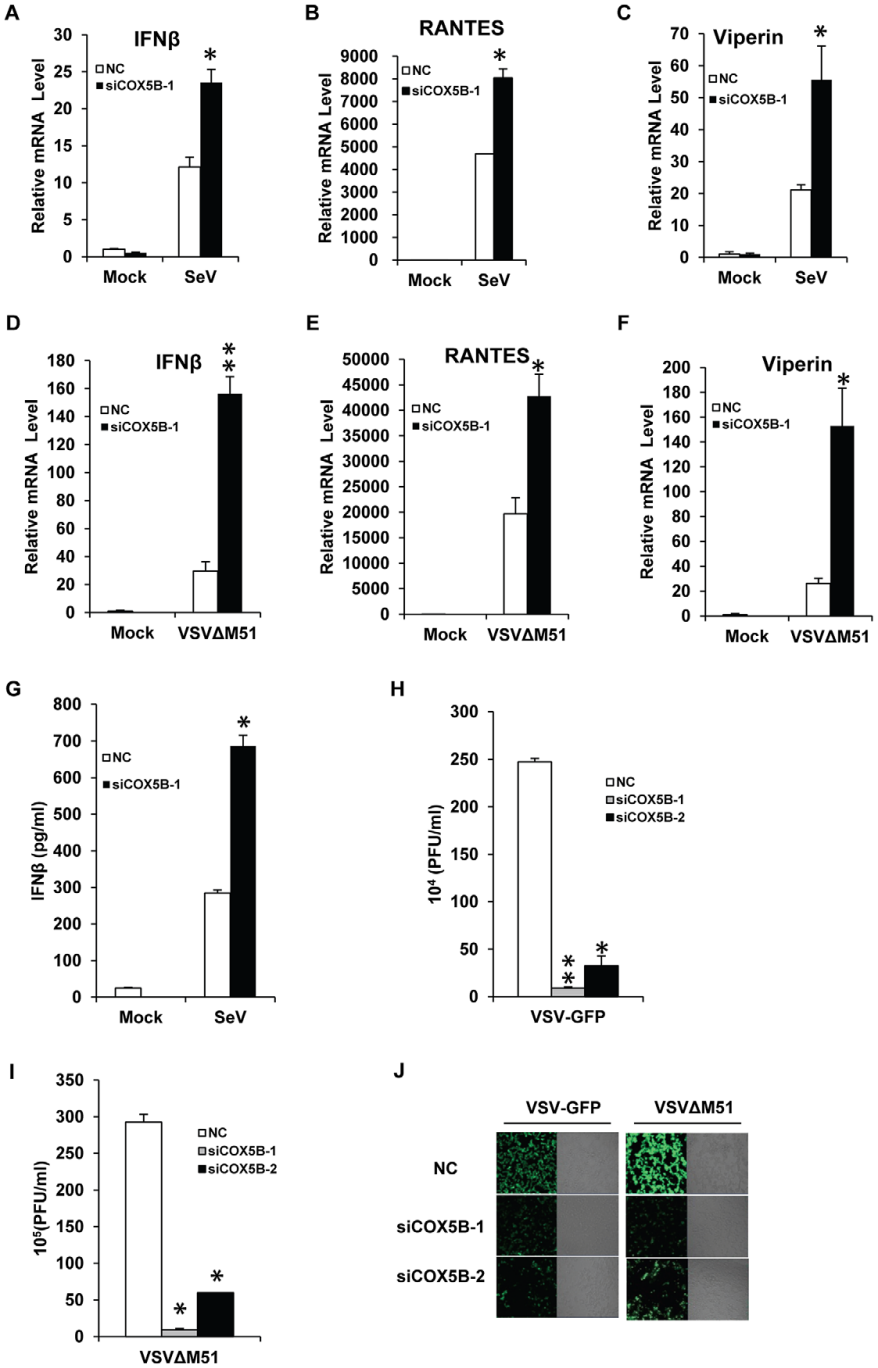
COX5B negatively controls the virus amplification. (A–C) HEK293 cells were transfected with COX5B RNAi oligos or NC. After 48 h transfection, cells were infected by Sendai virus for 12 h, and then lysed to isolate RNA to check the transcription levels of IFNβ (A), RANTES (B) and Viperin (C) by real-time PCR. (D–F) The transfection were performed as in (A–C), except that cells were infected by VSVΔM51-GFP (MOI = 0.1) for 9 h. (G) The RNAi oligo transfection was carried out as in (A), RNAi cells were infected by Sendai virus for 16 h, and the supernatant was collected for measurement of IFNβ by ELISA. (H–J) HEK293 cells were transfected with COX5B RNAi oligos for 48 h, then cells were infected by VSV-GFP (H) or VSVΔM51-GFP (I) at the MOI of 0.01 for 12 h, subsequently the culture supernatants were collected to measure virus titer by plaque assay, or cells were imaged by fluorescence microscopy (J). Data from A–I are representative of at least three independent experiments, (A–F, mean and s.d. of triplicate assays, G–I using duplicate assays). *, P<0.05; **, P<0.01 versus the control groups.

We next sought to determine the biological importance of COX5B in the antiviral process, particularly in controlling virus amplification. Given that the productions of IFNs are important for the host to fight against viruses [3], [4], we first assessed whether endogenous COX5B is involved in induction of IFN-β production upon virus infection. As shown in Figures 4A–4F, the mRNA levels of IFN-β, RANTES and Viperin induced by Sendai virus or VSVΔM51 were significantly increased when COX5B was knocked down in HEK293 cells, compared with the cells that were treated with control oligos. Similar results were obtained when COX5B knockdown stable RAW264.7 cells were infected with Sendai virus, compared with the control cells (Figures S5A–S5C in Text S1). In line with these findings, the protein level of IFN-β induced by Sendai virus was also increased in the absence of COX5B as measured by ELISA (Figure 4G). We then examined whether COX5B could mediate immune defense against the VSV-GFP and VSVΔM51. As shown in Figures 4H–4J, the levels of viral titer in the culture supernatants were significantly lower in HEK293 COX5B knockdown cells compared with control. Similar results were also obtained when another cell line, A549, was investigated (Figures S5D–S5E in Text S1). These findings together reveal that COX5B has biological functions in controlling antiviral signaling through interaction with MAVS.

### COX5B mediates MAVS signaling through repressing ROS production

**Figure 5:**
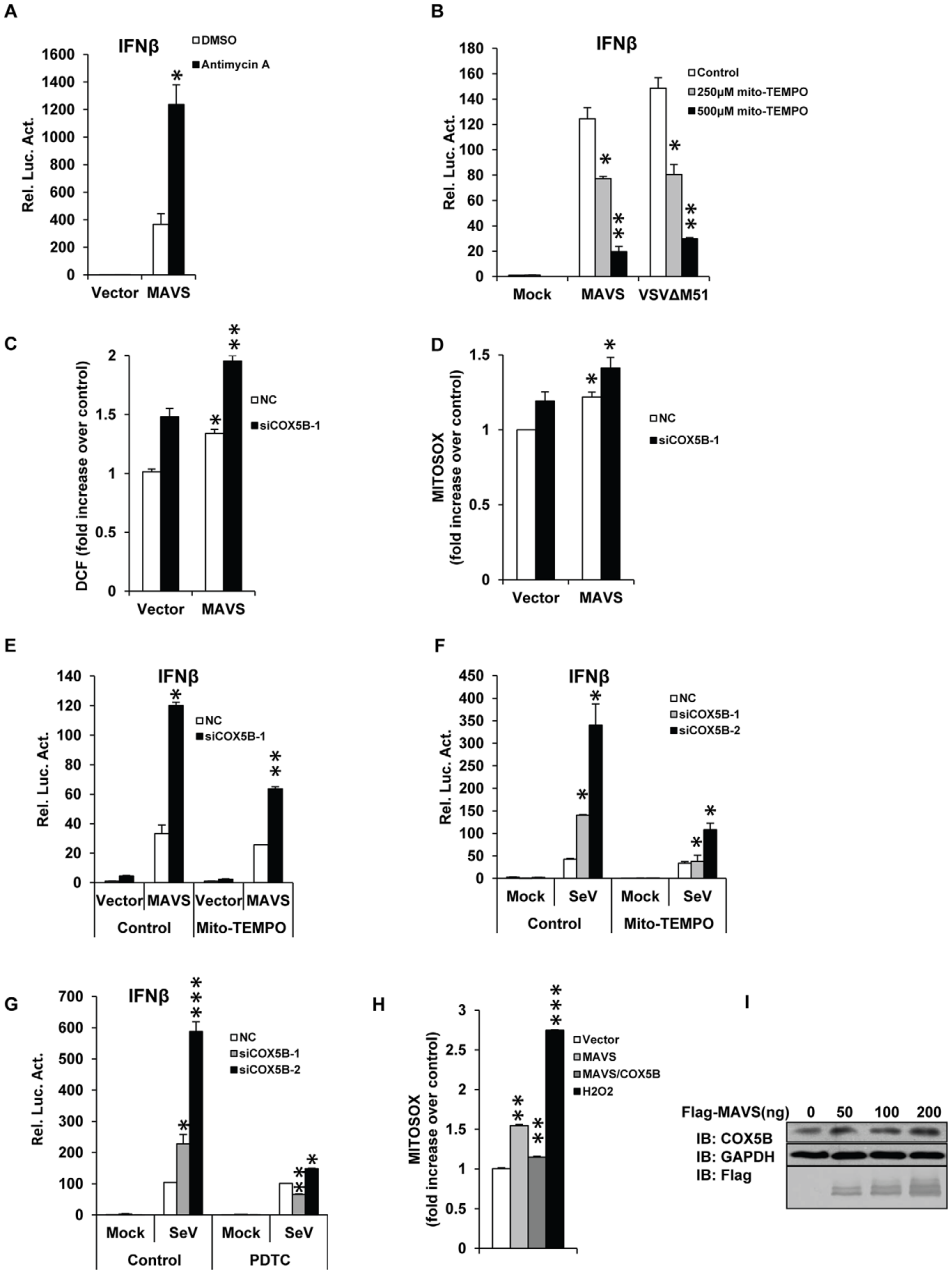
COX5B mediates MAVS signaling by repressing ROS production. (A) HEK293 cells were pretreated with 10 ug/ml antimycin A for 2 h, then transfected with the indicated plasmids. Twenty-four hours after transfection, cells were lysed to measure the IFNβ induction. (B) HEK293 cells were pretreated with 250 or 500 µM Mito-TEMPO for 4 h, and then transfected with IFNβ reporter and pRSV/LacZ vectors together with empty vector or MAVS, or infected with VSVΔM51-GFP (MOI = 0.1). Cells were lysed to measure IFNβ activity after 24 h transfection or 10 h infection. (C and D) COX5B RNAi oligos or NC were transfected into HEK293 cells, after 20 h transfection, empty vector or MAVS plasmids were transfected again, 30 h after the second transfection, cells were collected for FACS analysis to check cellular or mitochondrial ROS production by staining with DCF (C) or MitoSOX (D), respectively. Results are presented relative to the FACS mean fluorescence intensity over control cells. (E) HEK293 cells were transfected with COX5B RNAi oligo or NC, 36 h after transfection, cells were pretreated with 250 µM Mito-TEMPO for 4 h, and transfected again with indicated plasmids. Twenty-four hours after second transfection, cells were lysed to measure the IFNβ induction. (F) After first transfection and treatment as described in (E), cells were transfected with IFNβ reporter and pRSV/LacZ vectors, followed by 16 h Sendai virus infection, cells were harvested for luciferase assays. (G) The experiments were carried out as in (F) except that cells were pretreated with 100 µM PDTC. (H) HEK293 cells were transfected with the indicated plasmids for 30 h, and then stained with MitoSOX followed by FACS analysis. Cells were treated with 0.1 µM H_2_O_2_ for 30 min as positive control. Results are presented relative to the FACS mean fluorescence intensity in control cells. (I) HEK293 cells were transfected with increasing amounts of MAVS expression plasmids, and empty vector was used to balance the total DNA amount. Total protein was prepared and subjected to immunoblot analysis after 24 h transfection. Data from A–B, E–G are representative of at least three independent experiments, (mean and s.d. of duplicate assays), and data from C, D and H are presented as mean ± SD from at least three independent experiments. *, P<0.05; **, P<0.01; *** P<0.001 versus control groups.

We next sought to determine the molecular mechanism underlying the action of COX5B in the MAVS-mediated antiviral pathway. Cytochrome c oxidase (CcO) is a multi-subunit bigenomic protein complex which catalyzes the last step of the mitochondrial electron transport chain [21]. Among the complex, the subunit COX5B is a peripheral subunit of CcO complex [26]. Previous studies have shown that loss of COX5B resulted in dysfunction of mitochondria in cultured cells, in particular it increased the production of reactive oxygen species (ROS), revealing that in addition to its role in mitochondrial electron transport, COX5B is also required for oxygen tolerance [27]. To assess the role of COX5B in mitochondrial oxygen tolerance to MAVS-mediated antiviral signaling, we used an inducer of mitochondrial ROS, Antimycin A [28]–[30], to pre-treat cells before MAVS overexpression, and found that the treatment by Antimycin A evidently potentiated the activity of MAVS signaling (Figure 5A). Conversely, the treatment of Mito-TEMPO, a scavenger specific for mitochondrial ROS [31]–[33], attenuated the IFNβ promoter activity induced either by overexpression of MAVS or infected with VSVΔM51 virus (Figure 5B). Given that inactivation of COX5B enhances MAVS signaling activity and accordingly increases cellular and mitochondrial ROS levels (Figures 5C–5D), we tried to determine whether the increase of MAVS signaling activity by inactivation of COX5B is attributed to the increase of ROS levels. As shown in Figures 5E–5F, Mito-TEMPO treatment was sufficient to suppress the apparent increase in the levels of MAVS signaling activity by COX5B knockdown in MAVS-overexpressing cells, or in cells infected with Sendai virus. Consistent with this, we obtained similar results when another ROS inhibitor, PDTC [19], was used (Figure 5G).

Since ROS is involved in MAVS signaling activity, we then tested whether overexpression of MAVS itself affects the levels of ROS measured by using different ROS indicators. As shown in Figure 5H and Figure S6A–S6B in Text S1, overexpression of MAVS indeed increased ROS production compared with control, while overexpression of COX5B inhibited the increased ROS by MAVS. These results suggest that, in addition to the major role of MAVS in activating antiviral immune response, MAVS is also involved in altering the levels of ROS through interaction with COX5B. Notably, we found that overexpression of MAVS increased the levels of COX5B protein expression, but did not affect the levels of COX5B mRNA (Figures 5I and S7A in Text S1). In addition, we did not observe the up-regulation of COX5B expression when cells were treated with the purified IFNβ protein (Figure S7B in Text S1), suggesting that up-regulation of COX5B induced by activated MAVS signaling is independent on the IFNβ production. Given that COX5B is essential for ROS production and physically interacts with MAVS, our results support the concept that COX5B negatively regulates MAVS signaling through repressing ROS production.

### COX5B coordinates with ATG5 to repress MAVS signaling

**Figure 6:**
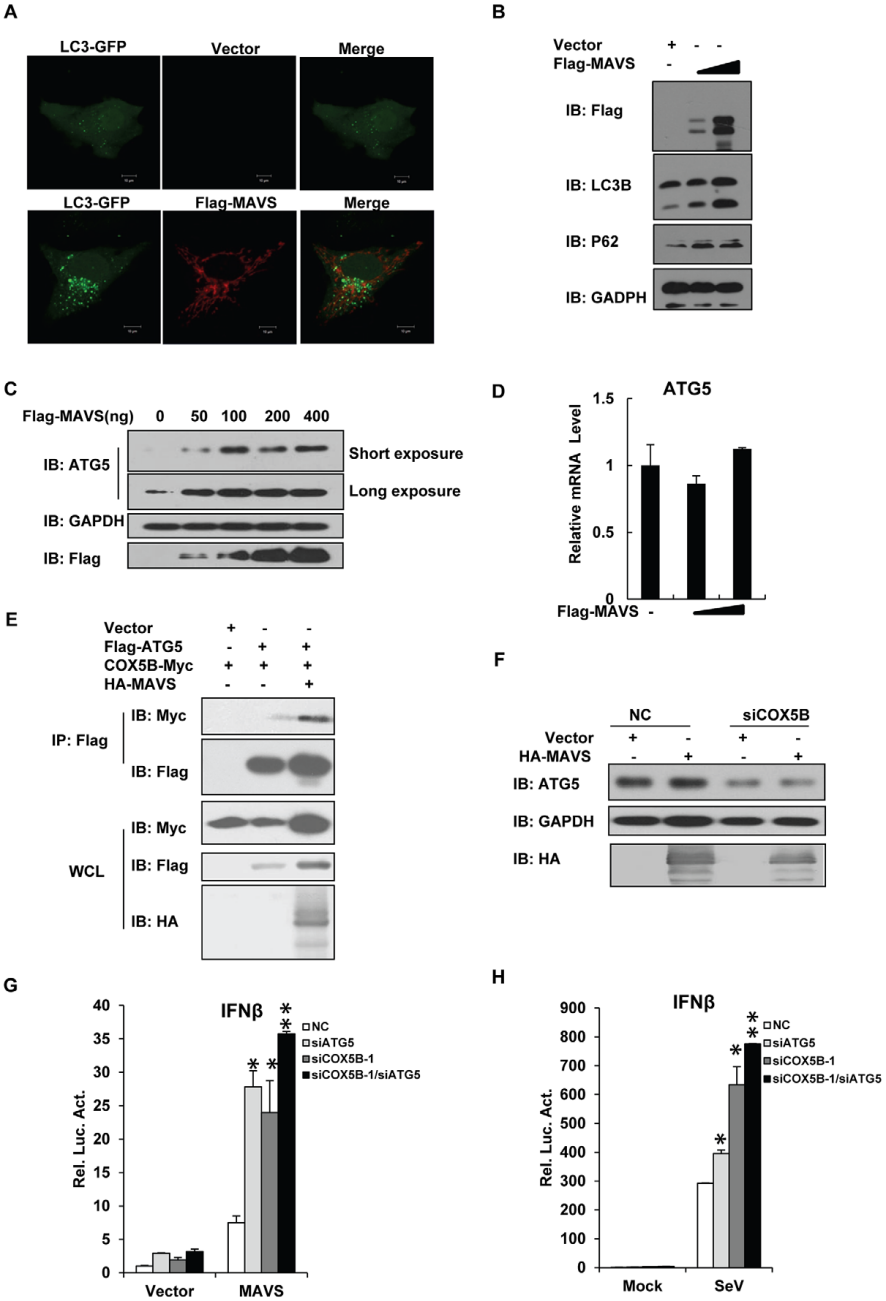
COX5B coordinates with ATG5 to regulate MAVS signaling. (A) Hela cells were transfected with LC3-GFP (Green) and control vector or Flag-MAVS (Red) for 24 h. Cells were fixed, then stained with anti-Flag antibody, and imaged by confocal microscopy. (B) Hela cells were transfected with increasing amounts of MAVS expression plasmids, and empty vector was used to balance the total DNA amount. Total protein was prepared and subjected to immunoblot analysis after 36 h transfection. (C) HEK293 cells were transfected with increasing amounts of MAVS expression plasmids, and empty vector was used to balance the total DNA amount. Twenty-four hours after transfection, total protein was extracted and subjected to immunoblot analysis. (D) Cell transfection described as seen in (C), after transfection, the total RNAs were prepared for real-time PCR analysis. (E) HEK293 cells were transfected as indicated expression plasmids. Twenty-four hours after transfection, cell lysates were prepared, immunoprecipitated with anti-Flag beads, followed by immunoblot analysis with the indicated antibodies. (F) HEK293 cells were transfected COX5B oligo or NC, after 30 h, transfected again with MAVS or empty vector plasmids, 24 h later, cells were lysed for western blotting. (G) ATG5 and COX5B RNAi oligos (final concentration 20 nM) were transfected into HEK293 cells, NC oligos were used to balance the equal amount of RNAi oligos. Thirty-six hours after transfection, cells were transfected again with the expression plasmids for MAVS or empty vector along with the reporter plasmids followed by measurement of luciferase assays after 24 h transfection. (H) ATG5 and COX5B RNAi oligo transfection was performed as described in (G), and then cells were transfected again with reporter plasmids. Eight hours after second transfection, cells were infected with Sendai virus for 16 h followed by measurement of luciferase assays. Data from D, G and H are representative of at least three independent experiments (mean and s.d. of duplicate assays). *, P<0.05; **, P<0.01 versus control groups.

Autophagy is an evolutionarily conserved cellular process that maintains cell homeostasis by removing damaged organelles and aggregated proteins [34]–[35]. Previous studies have shown that absence of autophagy amplifies antiviral signaling [18], [36], [37], [38], [39]. Given that the production of ROS is highly associated with autophagy, and negative roles of both autophagy and COX5B in controlling RLR signaling pathway, we reasoned that COX5B might coordinate with the autophagy pathway to control MAVS signaling. To test this hypothesis, we first examined whether overexpression of MAVS causes any change in the levels of autophagy. As shown in an immunostaining assay (Figure 6A), overexpression of MAVS evidently induced LC3 puncta formation when autophagy indicator, LC3-GFP, was used. The further western blot experiments showed that the activated MAVS not only led to increased LC3B type II formation, but also affected expression levels of P62 and ATG5 proteins (Figures 6B–6C). These findings together support a notion that activation of MAVS likely induces autophagy.

ATG5 is an essential component in the autophagy pathway, since loss of ATG5 completely blocks the autophagy process. We noted that, in contrast to the treatment of the purified IFNβ protein, overexpression of MAVS sufficiently elevated the protein levels (but not mRNA) of both COX5B and ATG5, (Figures 5I, 6C, 6D, S7A and S7B in Text S1). We then sought to test whether COX5B form a complex with ATG5 upon activation of MAVS signaling. As shown in Figure 6E, no apparent interaction between COX5B and ATG5 was observed when cells were transfected with COX5B and ATG5. However, the association of COX5B with ATG5 was easily detected when cells were also co-overexpressed with MAVS. These findings suggest that COX5B interacts with ATG5 likely in response to the stimulation of MAVS activation. We next asked whether COX5B regulates MAVS signaling via affecting ATG5 expression. As shown in Figure 6F, knockdown of COX5B markedly down-regulated ATG5 expression induced by activation of MAVS, suggesting that stabilization of ATG5 by MAVS depends on COX5B. Consistent with this, we also found that double knockdown of COX5B and ATG5 did not significantly increase MAVS signaling activity compared with knockdown of either COX5B or ATG5 alone in MAVS overexpressing cells, or in Sendai virus-infected cells (Figures 6G–6H). Collectively, these findings suggest that COX5B likely coordinates with ATG5 to regulate MAVS signaling in a common pathway.

### COX5B and ATG5 suppress MAVS aggregation to balance the MAVS signaling

**Figure 7:**
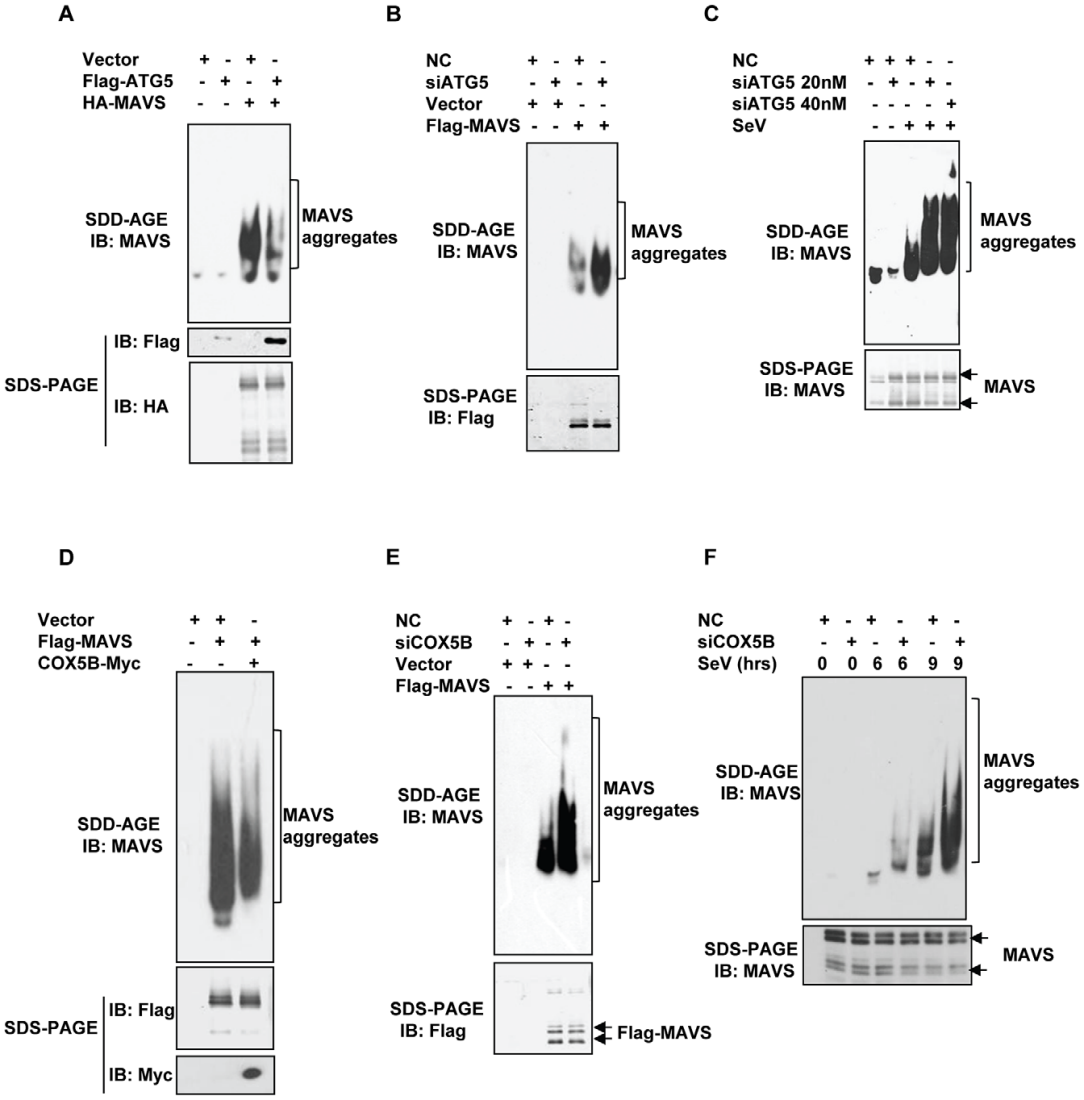
COX5B and ATG5 suppress MAVS aggregation. (A) HEK293 cells were transfected with the indicated constructs for 36 h, and crude mitochondrial P5 extracts were isolated, then aliquots of the P5 extracts were subjected to SDD-AGE and SDS-PAGE assays using indicated antibodies respectively. (B) HEK293 cells were transfected with NC or ATG5 RNAi oligos. Thirty-six hours after transfection, Flag-MAVS or empty vectors were transfected into the RNAi cells for 24 h. Crude mitochondrial P5 extracts were prepared, followed by SDD-AGE and SDS-PAGE assays using MAVS or Flag antibody. (C) HEK293 cells were transfected with NC or different doses of ATG5 RNAi oligos as indicated. Thirty-six hours after transfection, knockdown cells were then infected with Sendai virus, 9 h after infection, crude mitochondrial P5 extracts were analyzed by SDD-AGE or SDS-PAGE assays using a MAVS antibody. (D) Cell transfection was performed with indicated plasmids, Thirty-six hours after transfection, crude mitochondrial P5 extracts were isolated, and then aliquots of the P5 extracts were subjected to SDD-AGE and SDS-PAGE assays using indicated antibodies respectively. (E) The experiments were performed as in (B), except that COX5B RNAi oligos were used in lieu of ATG5 RNAi oligos. (F) HEK293 cells were transfected with NC or COX5B RNAi oligos as indicated. Thirty-six hours after transfection, knockdown cells were then infected with Sendai virus, six or nine hours after infection, cell lysates were analyzed by SDD-AGE or SDS-PAGE assays using a MAVS antibody.

It has been shown recently that viral infection efficiently induces MAVS conformational switch that leads to the formation of very large MAVS aggregates (also shown in Figure S8A in Text S1 in this study), which potently activate IRF3 and propagate the antiviral signaling [40]. Since autophagy is involved in removing aggregated proteins, we sought to test whether ATG5 regulates MAVS signaling by controlling MAVS aggregation. To test this possibility, we employed the immunostaining assay and semi-denaturing detergent agarose gel electrophoresis (SDD-AGE) analysis, a method described previously [40]–[41]. We detected the behavior of MAVS aggregation from control, ATG5 overexpression and ATG5 knockdown cells that were also transfected with MAVS (Figure S8B in Text S1). As shown in SDD-AGE assays (Figures 7A–7B), reduction and enhancement of MAVS aggregations were observed in overexpressing MAVS cells with ATG5 overexpression and ATG5 knockdown, respectively. Additionally, knockdown of ATG5 in HEK293 cells apparently increased the aggregation of endogenous MAVS protein induced by Sendai virus infection (Figure 7C). Similarly, MAVS aggregations (YFP-MAVS protein cluster formation) appeared to be enhanced in *Atg5*-deficient MEF cells compared to the wild-type MEF cells with expressing YFP-MAVS under microscopy (Figure S8C in Text S1). In addition, we also observed the enhancement of MAVS aggregations in ATG5 knockdown Hela cells with overexpressing MAVS or Sendai virus infection under microscopy by immunostaining (Figures S9A–S9B in Text S1). Thus, our results suggest that ATG5 functions as a negative regulator in MAVS signaling by controlling MAVS aggregation.

Given that COX5B acts in a common pathway with ATG5 in the regulation of MAVS signaling, we reasoned that COX5B might also be involved in regulating MAVS aggregation. As shown in Figures 7D–7E, while overexpression of COX5B reduced MAVS aggregates, knockdown of COX5B increased MAVS aggregates. Consistent with the above results, knockdown of COX5B in HEK293 cells apparently increased the aggregation of endogenous MAVS protein induced by Sendai virus infection (Figure 7F). Additionally, similar results were observed in COX5B knockdown Hela cells with overexpressing MAVS or Sendai virus infection in an immunostaining assays (Figures S9A–S9B in Text S1). Taken together, our results support the concept that COX5B and ATG5 act in a common pathway to negatively regulate MAVS aggregation.

## Discussion

MAVS plays the central role in RLR receptors-mediated anti-virus signaling pathway. Previous studies have identified several MAVS-associated factors such as MFN2 [15], NLRX1 [11], PCBP2 [42] and PLK1 [43] negatively regulate MAVS function to maintain immune balance. However, the mechanism underlying the regulatory relationship between MAVS and mitochondria still remains not well-understood. In this study, we have identified that a subunit of the CcO complex, COX5B, physically interacts with MAVS, and functions as a negative regulator in MAVS-mediated antiviral signaling pathway. We found that COX5B mediates MAVS signaling by repressing ROS production. Importantly, we provided evidence to support that COX5B functions in concert with ATG5 to suppress MAVS aggregation, thereby balancing MAVS signaling upon virus challenge. Thus, our study uncovers a novel mechanism by which the mitochondrial CcO component COX5B coordinates with the autophagy pathway to negatively regulate antiviral signaling.

The mitochondrion is an essential organelle of eukaryotic cells that generates adenosine triphosphate (ATP) for energy production [44]. COX5B is an important subunit of the CcO complex that functions as the terminal enzyme of the mitochondrial electron transport chain [21], [45], [46]. In addition to its role in the transfer of electrons from reduced cytochrome c to molecular oxygen, COX5B has been shown to play a role in suppressing ROS production [21], [27]. Increasing pieces of evidence have linked the roles of ROS in the regulation of antiviral signaling and mitochondrion homeostasis as well [17]–[19]. However, the molecular basis by which ROS regulates antiviral signaling remains poorly understood. Identification of the CcO complex COX5B as a MAVS-interacting factor motivates us to investigate the detailed regulatory mechanism between MAVS-mediated antiviral signaling and COX5B, since COX5B plays an important role for regulating ROS production, in addition to its role in maintaining CcO complex activity [21]. Our study reveals that activation of MAVS not only stimulates the antiviral response, but also results in increased levels of ROS production. Interestingly, we found that activation of MAVS appears to stabilize COX5B protein, which represses ROS production and consequently down-regulates the antiviral response in a feedback regulatory fashion. Thus, on the one hand, upon virus challenges, MAVS causes mitochondria to induce ROS production and activate antiviral signaling. On the other hand, MAVS elevates COX5B expression to suppress ROS, thereby balancing antiviral signaling activity to a proper level and likely ensuring mitochondrion homeostasis.

As mentioned above, COX5B severs as a mitochondrial CcO component and has been proposed to be predominantly localized to the matrix face of the mitochondrial inner membrane [47]. Thus, the question becomes as to how COX5B interacts with MAVS, a mitochondrial outer membrane associated protein in physiological condition. It has been shown previously that COX5B interacts with human androgen receptor (hAR), which is present outside mitochondria [48]. Consistent with this, we have indeed detected a low level of COX5B protein present in the cytosolic compartment that was confirmed by our IP experiments (Figure 1F), since a weak binding was detected between COX5B and MAVSΔTM, which is localized to cytosolic compartment. Upon activation of MAVS signaling, activated MAVS appeared to increase the total COX5B protein levels (Figure 5I). Given that activation of MAVS could change morphology of mitochondrion [40] and reduce its membrane potential (loss of Δψ_m_) [16], [49], it is thus likely that activation of MAVS increases the levels of cytosol-COX5B fraction and enhances the interaction of cytosol-COX5B with MAVS. In fact, several lines of evidence in this study support this notion. First, the activated MAVS indeed induces a high level of cytosolic fraction of COX5B (Figure S10A in Text S1). Second, the activated MAVS enhances the interaction of COX5B with MAVSΔTM, which is predominantly cytosolic region (Figure S10B in Text S1). Third, activation of MAVS promotes the interaction of COX5B with ATG5, which is also present in cytosolic compartment.

We noted that the mutant form of COX5B, COX5BΔTP, failed to inhibit MAVS signaling (Figure 2I–2J), suggesting mitochondrial targeting is important for the role of COX5B in the suppressing MAVS signaling. To address this issue, we generated another mutant form of COX5B-TM (Bcl-2), in which the COX5BΔTP mutant was modified by adding the transmembrane domain (the TM from Bcl2) at the C-terminal, for targeting COX5B-TM (Bcl-2) to mitochondrial outer membrane. As shown in Figures S11A–S11B in Text S1, expression of COX5B-TM (Bcl-2) is sufficient to suppress the MAVS signaling in transfected cells, emphasizing the potential biological importance of COX5B at mitochondrial outer membrane.

Autophagy is a conserved process for cell homeostasis which removes aggregated proteins and dysfunctional organelles. Previous studies have proposed that autophagy is also engaged in regulating anti-virus signaling, since loss of autophagy components (e.g. ATG5 or ATG12) causes amplification of MAVS-mediated antiviral signaling [18], [36]. Thus, the important question that remains to be determined is how autophagy regulates the antiviral signaling pathway. In this study, we showed that activation of MAVS up-regulates the expression of both COX5B and ATG5 proteins, and COX5B forms a complex with ATG5 in a MAVS-activation dependent manner. Moreover, we also found that stabilization of ATG5 by activation of MAVS depends on COX5B. Thus, our data indicate that COX5B and ATG5 modulate the MAVS function in a common regulatory pathway. A recent study has shown that viral infection leads to the formation of very large MAVS aggregates, which potently activates MAVS-mediated antiviral signaling. Given that the autophagy pathway normally functions in degrading protein aggregates [34]–[35], we therefore determined whether the autophagy pathway regulates MAVS aggregates. Indeed, we found that ATG5 plays an important role in controlling aggregation of MAVS, since loss of ATG5 increases formation of MAVS aggregates, while overexpression of ATG5 reduces MAVS aggregates. Surprisingly, we observed that COX5B has a similar function as ATG5 does in controlling aggregation of MAVS. Given that COX5B also affects the stability of ATG5, our results suggest that COX5B might maintain the proper function of autophagy in regulating MAVS signaling.

In conclusion, in this study we have identified that in addition to its role in the transfer of electrons in mitochondria, the CcO complex subunit COX5B also has a novel function in regulating the antiviral pathway by interacting with MAVS. Importantly, our findings suggest that COX5B represses mitochondrial ROS production and coordinates with the autophagy pathway to control MAVS aggregation, thereby balancing the antiviral signaling activity. Thus, our study not only reveals the novel role of CcO components in regulating antiviral signaling, but also provides novel insights into the link between mitochondrial electron transfer machinery and the autophagy pathway in controlling ROS production and host innate immunity.

## Materials and Methods

### Cells, antibodies, viruses and reagents

HEK293, Hela, A549, HepG2, RAW264.7, Vero and MEF cells were cultured with high-glucose DMEM (Hyclone) medium plus 10% heat-inactivated FBS (Hyclone), and supplemented with antibiotics (100 unit/ml penicillin, 100 µg/ml streptomycin, Gibco). The antibody against MAVS was generated by immunizing rabbits with the recombinant proteins His_6_-MAVS (131–291) produced in *E. coli*. Antibody was further purified using an antigen column. The antibodies against COX5B, IRF3 and COX4 were purchased from Santa Cruz Biotechnology. Other antibodies used were as follows: mouse anti-flag M2 (Sigma); rabbit anti-Myc (MBL); mouse anti-HA (Covance); mouse anti-GAPDH (Sungene Biotech); mouse anti-mouse-COX5B (Abcam); rabbit anti-ATG5 (Epitomics), rabbit anti-AIF (CST), rabbit anti-α tubulin (Abcam), rabbit anti-LC3B (MBL), rabbit anti-P62 (Epitomics). Sendai virus (Cantell strain, Charles River Laboratories) was used at a final concentration of 50 hemagglutinating (HA) units/ml. VSV-GFP [50] and VSVΔM51-GFP [24] was propagated in Vero cells. TNFα was purchased from Sigma. ROS-related reagents used here as Mito-TEMPO (Santa Cruz, Enzo Life Science), Amplex Red Hydrogen Peroxide/Peroxidase Assay Kit (Molecular Probes, Invitrogen), Antimycin A (Sigma), PDTC (Sigma) were purchased from indicated suppliers. The ELISA kits for Human IFN-β were purchased from PBL Biomedical Laboratories.

### Plasmids

Human full length COX5B, truncated mutant, COX5A and ATG5 were amplified from human fetal liver cDNA library through standard PCR methods, and then cloned into pcDNA3.1-myc/his (-) A, pEGFP-N3 or pcDNA3.0-Flag vectors respectively. Plasmids of Flag-MAVS, HA-MAVS, Flag-RIG-I(N), Trif, NF-κB-Luc, pET14b-MAVS (131–291) were generously provided by Dr. Zhijian Chen. IFNβ-Luc and ISRE-Luc reporters were kindly provided by Dr. Hongbing Shu [51]. MAVS lacking CARD domain (10–77) and TM domain (514–540) were cloned into pcDNA3.0 vector using overlap cloning techniques. Full length MAVS was subcloned into pEYFP-C1 vector. pSUPERIOR-4*sh-mouse-COX5B vector construction was carried out as previously described [52], target sequence for shRNA mCOX5B is as follows, GAGGACAACUGUACUGUCA [21].

### Yeast two-hybrid screening

To construct a MAVS bait vector, a cDNA fragment encoding the CARD domain of MAVS was inserted in-frame into pGBKT7 vector. A human fetal liver cDNA library was screened according to protocols recommended by the manufacturer (Clontech).

### Luciferase reporter assay

HEK293 cells were transfected in a 24-well plate with various expression plasmids along with a reporter plasmid (50 ng/well) and a pRSV/LacZ (50 ng/well) using the calcium phosphate precipitation method. Twenty-four hours post-transfection, cells were lysed for the measurement of luciferase activity. Firefly luciferase activities were normalized based on the β-galactosidase activity.

### RNAi

HEK293 cells were transfected with RNAi oligos at a final concentration of 40 nM using the calcium phosphate precipitation method. Forty-eight hours later, cells were transfected with indicated plasmids using lipofectamine 2000 (Invitrogen) or stimulated with virus, then harvested for the further reporter, western blot or real-time RCR analysis, unless indicated otherwise. HepG2 and A549 cells were transfected with siRNA oligos using Lipofectamine RNAiMAX (Invitrogen) reagent. The RNAi sequences are as follows (only the sense strand is shown): NC (non-targeting), TTCTCCGAACGTGTCACGT; GFP, GCAGAAGAACGGCATCAAG; COX5B-1, GCATCTGTGAAGAGGACAA; COX5B-2, GGGACTGGACCCATACAAT; COX5B-3, CAGTAAAGACTAGCCATTG [27]; MAVS, CCACCTTGATGCCTGTGAA [6]; ATG5, GATTCATGGAATTGAGCCA.

### Real-time PCR

Total RNA was isolated using TRIZOL reagent (Invitrogen). cDNA was synthesized using SuperScript III First-Strand cDNA Synthesis kit (Invitrogen). Real-time PCR was performed using SYBR Premix Ex Taq (TARKRA) in triplicate on a Bio-Rad iCycler iQ5 PCR Thermal Cycler.

The primers used were as follows (5′-3′):

18S-s: 5′-GCTGCTGGCACCAGACTT-3′,

18S-as: 5′-CGGCTACCACATCCAAGG-3′;

GAPDH-s: 5′-ATGACATCAAGAAGGTGGTG-3′,

GAPDH-as: 5′-CATACCAGGAAATGAGCTTG-3′;

IFNβ-s: 5′-AGGACAGGATGAACTTTGAC-3′,

IFNβ-as: 5′-TGATAGACATTAGCCAGGAG-3′;

Viperin-s: 5′-CTTTGTGCTGCCCCTTGAGGAA-3′,

Viperin-as: 5′-CTCTCCCGGATCAGGCTTCCA-3′;

RANTES-s: 5′-TACACCAGTGGCAAGTGCTC-3′,

RANTES-as: 5′-TGTACTCCCGAACCCATTTC-3′;

IFNβ (mouse)-s: 5′-ATGGTGGTCCGAGCAGAGAT-3′;

IFNβ (mouse)-as: 5′-CCACCACTCATTCTGAGGCA-3′;

Actin (mouse)-s: 5′-TCCAGCCTTCCTTCTTGGGT-3′,

Actin (mouse)-as: 5′-GCACTGTGTTGGCATAGAGGT-3′;

RANTES (mouse)-s: 5′-CTCACCATATGGCTCGGACA-3′,

RANTES (mouse)-as: 5′-ACAAACACGACTGCAAGATTGG-3′ [53].

### Coimmunoprecipitation and immunoblot analysis

For coimmunoprecipitation assays, cells were collected 24 h after transfection and then lysed in 0.5% Triton X-100 buffer (20 mM Tris, pH 7.5, 150 mM NaCl, 0.5% Triton X-100, 1 mM EDTA, 10% glycerol, 10 µg/ml aprotinin, 10 µg/ml leupeptin, 1 mM phenylmethylsulfonyl fluoride) on ice for 45 min, sonicated briefly, then the clarified supernatants were incubated with anti-Flag ( Sigma) agarose beads for 4 h or with anti-Myc antibody overnight at 4°C followed by further incubation with protein A/G beads (Pierce) for 2–4 h. The immune complexes were washed with lysis buffer three times and subjected to immunoblot analysis with the indicated antibodies. For endogenous IP, HEK293T cells were lysed with 1% NP-40 buffer, sonicated briefly, and the supernatants were incubated with MAVS antibody or control IgG overnight at 4°C, followed by further incubation with protein A/G beads (Pierce) for 2–4 h. Immunoblotting was carried out by standard procedures.

### Confocal imaging

For all microscopy images, cells were grown on glass coverslips and transfected, as described. After washing with PBS, cells were fixed with 4% paraformaldehyde for 15 minutes, permeabilized and blocked with 0.2% Triton X-100 in PBS containing 5% BSA for 30 minutes at room temperature. Cells were then incubated with primary antibody and secondary antibody or mounted onto slides directly. Imaging of the cells was performed using Zeiss LSM 710 META laser scanning confocal system.

### Native gel electrophoresis and semidenaturing detergent agarose gel electrophoresis

Native gel electrophoresis and IRF3 dimerization assays were performed as described previously [50]. Semidenaturing detergent agarose gel electrophoresis (SDD-AGE) was carried out according to a published protocol [40]–[41]. Briefly, HEK293 cells were homogenized in a buffer (10 mM Tris-HCl [pH 7.5, 10 mM KCl, 1.5 mM MgCl2, 0.25 M D-mannitol, and Roche EDTA-free protease inhibitor cocktail) by repeated douncing. Crude mitochondria (P5) were resuspended and loaded onto a vertical 1.5% agarose gel after purification by differential centrifugation. Electrophoresis was done as described [40]. Immunoblotting was carried out by standard procedures.

### Virus plaque assay

On the third day after knockdown of COX5B using RNAi oligo, HEK293 cells were infected with VSV-GFP or VSVΔM51-GFP at 0.01 MOI. The supernatants were collected at indicated times for measurement of virus titer by virus plaque assays. The assay was done as described [50] with minor modification. Briefly, confluent Vero cells were infected with the diluted virus for 1 h, then culture medium containing 2% methylcellulose was overlaid and incubated for about 36 h. Cells were fixed for 15 min with methanol and then stained with 1% crystal violet to display plaques. Plaques were counted to determine the viral titer as plaque-forming units per ml.

### ROS measurements and flow cytometric analysis

Cells were stained with MitoSOX (to measure the mROS superoxide) or CM-H_2_DCFDA (to measure total cellular ROS) (Molecular Probes, Invitrogen) at 5 µm or 2.5 µm final concentration for 30 minutes or 15 minutes at 37°C in dark, respectively. Cells were then washed with PBS solution, resuspended in PBS solution containing 1% FBS for FACS analysis. All data were analyzed with Cell Quest software (BD Biosciences).

### ELISA

Culture media was collected and analyzed for IFN production by using ELISA kit according to protocols recommended by the manufacturer (PBL Biomedical Laboratories).

### Statistical analyses

All statistical analyses are expressed as means ±SD. Significant differences between values under different experimental conditions were determined by two-tailed Student's *t*-test. For all test, a p value of less than 0.05 was considered statistically significant.

### NCBI accession numbers for genes used in this study

The mRNA sequence data for genes described in this study can be found in the NCBI under the following accession numbers: *Homo sapiens* COX5B (NM_001862), *Homo sapiens* COX5A(NM_004255), *Homo sapiens* MAVS (NM_020746), *Homo sapiens* P62 (NM_003900), *Homo sapiens* LC3B (NM_022818), *Homo sapiens* RIG-I (NM_014314), *Homo sapiens* MDA5(NM_022168), *Homo sapiens* ATG5(NM_004849).

## Supporting Information

Text S1 **Text S1 contains supplementary Figures S1 to S11 and one supplementary Table S1.**
(PDF)

## References

[1] Akira S, Uematsu S, Takeuchi O (2006) Pathogen recognition and innate immunity. Cell 124: 783–801. 16497588 10.1016/j.cell.2006.02.015

[2] Yoneyama M, Fujita T (2009) RNA recognition and signal transduction by RIG-I-like receptors. Immunol Rev 227: 54–65. 19120475 10.1111/j.1600-065X.2008.00727.x

[3] Baccala R, Kono DH, Theofilopoulos AN (2005) Interferons as pathogenic effectors in autoimmunity. Immunol Rev 204: 9–26. 15790347 10.1111/j.0105-2896.2005.00252.x

[4] Theofilopoulos AN, Baccala R, Beutler B, Kono DH (2005) Type I interferons (alpha/beta) in immunity and autoimmunity. Annu Rev Immunol 23: 307–336. 15771573 10.1146/annurev.immunol.23.021704.115843

[5] Onoguchi K, Yoneyama M, Takemura A, Akira S, Taniguchi T, et al (2007) Viral infections activate types I and III interferon genes through a common mechanism. J Biol Chem 282: 7576–7581. 17204473 10.1074/jbc.M608618200

[6] Seth RB, Sun L, Ea CK, Chen ZJ (2005) Identification and characterization of MAVS, a mitochondrial antiviral signaling protein that activates NF-kappaB and IRF 3. Cell 122: 669–682. 16125763 10.1016/j.cell.2005.08.012

[7] Kawai T, Takahashi K, Sato S, Coban C, Kumar H, et al (2005) IPS-1, an adaptor triggering RIG-I- and Mda5-mediated type I interferon induction. Nat Immunol 6: 981–988. 16127453 10.1038/ni1243

[8] Xu LG, Wang YY, Han KJ, Li LY, Zhai Z, et al (2005) VISA is an adapter protein required for virus-triggered IFN-beta signaling. Mol Cell 19: 727–740. 16153868 10.1016/j.molcel.2005.08.014

[9] Meylan E, Curran J, Hofmann K, Moradpour D, Binder M, et al (2005) Cardif is an adaptor protein in the RIG-I antiviral pathway and is targeted by hepatitis C virus. Nature 437: 1167–1172. 16177806 10.1038/nature04193

[10] Fitzgerald KA, McWhirter SM, Faia KL, Rowe DC, Latz E, et al (2003) IKKepsilon and TBK1 are essential components of the IRF3 signaling pathway. Nat Immunol 4: 491–496. 12692549 10.1038/ni921

[11] Moore CB, Bergstralh DT, Duncan JA, Lei Y, Morrison TE, et al (2008) NLRX1 is a regulator of mitochondrial antiviral immunity. Nature 451: 573–577. 18200010 10.1038/nature06501

[12] Arnoult D, Soares F, Tattoli I, Castanier C, Philpott DJ, et al (2009) An N-terminal addressing sequence targets NLRX1 to the mitochondrial matrix. J Cell Sci 122: 3161–3168. 19692591 10.1242/jcs.051193 PMC2871076

[13] Zhong B, Zhang L, Lei C, Li Y, Mao AP, et al (2009) The ubiquitin ligase RNF5 regulates antiviral responses by mediating degradation of the adaptor protein MITA. Immunity 30: 397–407. 19285439 10.1016/j.immuni.2009.01.008

[14] Onoguchi K, Onomoto K, Takamatsu S, Jogi M, Takemura A, et al (2010) Virus-infection or 5′ppp-RNA activates antiviral signal through redistribution of IPS-1 mediated by MFN1. PLoS Pathog 6: e1001012. 20661427 10.1371/journal.ppat.1001012 PMC2908619

[15] Yasukawa K, Oshiumi H, Takeda M, Ishihara N, Yanagi Y, et al (2009) Mitofusin 2 inhibits mitochondrial antiviral signaling. Sci Signal 2: ra47. 19690333 10.1126/scisignal.2000287

[16] Lei Y, Moore CB, Liesman RM, O'Connor BP, Bergstralh DT, et al (2009) MAVS-mediated apoptosis and its inhibition by viral proteins. PLoS One 4: e5466. 19404494 10.1371/journal.pone.0005466 PMC2674933

[17] Soucy-Faulkner A, Mukawera E, Fink K, Martel A, Jouan L, et al (2010) Requirement of NOX2 and reactive oxygen species for efficient RIG-I-mediated antiviral response through regulation of MAVS expression. PLoS Pathog 6: e1000930. 20532218 10.1371/journal.ppat.1000930 PMC2880583

[18] Tal MC, Sasai M, Lee HK, Yordy B, Shadel GS, et al (2009) Absence of autophagy results in reactive oxygen species-dependent amplification of RLR signaling. Proc Natl Acad Sci U S A 106: 2770–2775. 19196953 10.1073/pnas.0807694106 PMC2650341

[19] Gonzalez-Dosal R, Horan KA, Rahbek SH, Ichijo H, Chen ZJ, et al (2011) HSV infection induces production of ROS, which potentiate signaling from pattern recognition receptors: role for S-glutathionylation of TRAF3 and 6. PLoS Pathog 7: e1002250. 21949653 10.1371/journal.ppat.1002250 PMC3174249

[20] Tal MC, Iwasaki A (2009) Autophagic control of RLR signaling. Autophagy 5: 749–750. 19571662 10.4161/auto.5.5.8789 PMC3693554

[21] Galati D, Srinivasan S, Raza H, Prabu SK, Hardy M, et al (2009) Role of nuclear-encoded subunit Vb in the assembly and stability of cytochrome c oxidase complex: implications in mitochondrial dysfunction and ROS production. Biochem J 420: 439–449. 19338496 10.1042/BJ20090214 PMC2735414

[22] Hughes GJ, Frutiger S, Paquet N, Pasquali C, Sanchez JC, et al (1993) Human liver protein map: update 1993. Electrophoresis 14: 1216–1222. 8313870 10.1002/elps.11501401181

[23] Stojdl DF, Lichty BD, tenOever BR, Paterson JM, Power AT, et al (2003) VSV strains with defects in their ability to shutdown innate immunity are potent systemic anti-cancer agents. Cancer Cell 4: 263–275. 14585354 10.1016/s1535-6108(03)00241-1

[24] Zeng W, Xu M, Liu S, Sun L, Chen ZJ (2009) Key role of Ubc5 and lysine-63 polyubiquitination in viral activation of IRF3. Mol Cell 36: 315–325. 19854139 10.1016/j.molcel.2009.09.037 PMC2779157

[25] Iwamura T, Yoneyama M, Yamaguchi K, Suhara W, Mori W, et al (2001) Induction of IRF-3/-7 kinase and NF-kappaB in response to double-stranded RNA and virus infection: common and unique pathways. Genes Cells 6: 375–388. 11318879 10.1046/j.1365-2443.2001.00426.x

[26] Lomax MI, Hsieh CL, Darras BT, Francke U (1991) Structure of the human cytochrome c oxidase subunit Vb gene and chromosomal mapping of the coding gene and of seven pseudogenes. Genomics 10: 1–9. 1646156 10.1016/0888-7543(91)90476-u

[27] Campian JL, Gao X, Qian M, Eaton JW (2007) Cytochrome C oxidase activity and oxygen tolerance. J Biol Chem 282: 12430–12438. 17303578 10.1074/jbc.M604547200

[28] Diaz F, Enriquez JA, Moraes CT (2012) Cells lacking Rieske iron-sulfur protein have a reactive oxygen species-associated decrease in respiratory complexes I and IV. Mol Cell Biol 32: 415–429. 22106410 10.1128/MCB.06051-11 PMC3255782

[29] Park WH, Han YW, Kim SH, Kim SZ (2007) An ROS generator, antimycin A, inhibits the growth of HeLa cells via apoptosis. J Cell Biochem 102: 98–109. 17372917 10.1002/jcb.21280

[30] Hao L, Sakurai A, Watanabe T, Sorensen E, Nidom CA, et al (2008) Drosophila RNAi screen identifies host genes important for influenza virus replication. Nature 454: 890–893. 18615016 10.1038/nature07151 PMC2574945

[31] Trnka J, Blaikie FH, Logan A, Smith RA, Murphy MP (2009) Antioxidant properties of MitoTEMPOL and its hydroxylamine. Free Radic Res 43: 4–12. 19058062 10.1080/10715760802582183 PMC2645131

[32] Jiang J, Stoyanovsky DA, Belikova NA, Tyurina YY, Zhao Q, et al (2009) A mitochondria-targeted triphenylphosphonium-conjugated nitroxide functions as a radioprotector/mitigator. Radiat Res 172: 706–717. 19929417 10.1667/RR1729.1 PMC2804962

[33] Nakahira K, Haspel JA, Rathinam VA, Lee SJ, Dolinay T, et al (2011) Autophagy proteins regulate innate immune responses by inhibiting the release of mitochondrial DNA mediated by the NALP3 inflammasome. Nat Immunol 12: 222–230. 21151103 10.1038/ni.1980 PMC3079381

[34] Klionsky DJ, Emr SD (2000) Autophagy as a regulated pathway of cellular degradation. Science 290: 1717–1721. 11099404 10.1126/science.290.5497.1717 PMC2732363

[35] Mizushima N, Klionsky DJ (2007) Protein turnover via autophagy: implications for metabolism. Annu Rev Nutr 27: 19–40. 17311494 10.1146/annurev.nutr.27.061406.093749

[36] Jounai N, Takeshita F, Kobiyama K, Sawano A, Miyawaki A, et al (2007) The Atg5 Atg12 conjugate associates with innate antiviral immune responses. Proc Natl Acad Sci U S A 104: 14050–14055. 17709747 10.1073/pnas.0704014104 PMC1955809

[37] Saitoh T, Fujita N, Hayashi T, Takahara K, Satoh T, et al (2009) Atg9a controls dsDNA-driven dynamic translocation of STING and the innate immune response. Proc Natl Acad Sci U S A 106: 20842–20846. 19926846 10.1073/pnas.0911267106 PMC2791563

[38] Takeshita F, Kobiyama K, Miyawaki A, Jounai N, Okuda K (2008) The non-canonical role of Atg family members as suppressors of innate antiviral immune signaling. Autophagy 4: 67–69. 17921696 10.4161/auto.5055

[39] Lee HK, Lund JM, Ramanathan B, Mizushima N, Iwasaki A (2007) Autophagy-dependent viral recognition by plasmacytoid dendritic cells. Science 315: 1398–1401. 17272685 10.1126/science.1136880

[40] Hou F, Sun L, Zheng H, Skaug B, Jiang QX, et al (2011) MAVS forms functional prion-like aggregates to activate and propagate antiviral innate immune response. Cell 146: 448–461. 21782231 10.1016/j.cell.2011.06.041 PMC3179916

[41] Alberti S, Halfmann R, King O, Kapila A, Lindquist S (2009) A systematic survey identifies prions and illuminates sequence features of prionogenic proteins. Cell 137: 146–158. 19345193 10.1016/j.cell.2009.02.044 PMC2683788

[42] You F, Sun H, Zhou X, Sun W, Liang S, et al (2009) PCBP2 mediates degradation of the adaptor MAVS via the HECT ubiquitin ligase AIP4. Nat Immunol 10: 1300–1308. 19881509 10.1038/ni.1815

[43] Vitour D, Dabo S, Ahmadi Pour M, Vilasco M, Vidalain PO, et al (2009) Polo-like kinase 1 (PLK1) regulates interferon (IFN) induction by MAVS. J Biol Chem 284: 21797–21809. 19546225 10.1074/jbc.M109.018275 PMC2755906

[44] Attardi G, Schatz G (1988) Biogenesis of mitochondria. Annu Rev Cell Biol 4: 289–333. 2461720 10.1146/annurev.cb.04.110188.001445

[45] Kadenbach B, Huttemann M, Arnold S, Lee I, Bender E (2000) Mitochondrial energy metabolism is regulated via nuclear-coded subunits of cytochrome c oxidase. Free Radic Biol Med 29: 211–221. 11035249 10.1016/s0891-5849(00)00305-1

[46] Beauvoit B, Rigoulet M (2001) Regulation of cytochrome c oxidase by adenylic nucleotides. Is oxidative phosphorylation feedback regulated by its end-products? IUBMB Life 52: 143–152. 11798026 10.1080/152165401317316545

[47] Tsukihara T, Aoyama H, Yamashita E, Tomizaki T, Yamaguchi H, et al (1996) The whole structure of the 13-subunit oxidized cytochrome c oxidase at 2.8 A. Science 272: 1136–1144. 8638158 10.1126/science.272.5265.1136

[48] Beauchemin AM, Gottlieb B, Beitel LK, Elhaji YA, Pinsky L, et al (2001) Cytochrome c oxidase subunit Vb interacts with human androgen receptor: a potential mechanism for neurotoxicity in spinobulbar muscular atrophy. Brain Res Bull 56: 285–297. 11719263 10.1016/s0361-9230(01)00583-4

[49] Koshiba T, Yasukawa K, Yanagi Y, Kawabata S (2011) Mitochondrial membrane potential is required for MAVS-mediated antiviral signaling. Sci Signal 4: ra7. 21285412 10.1126/scisignal.2001147

[50] Sun Q, Sun L, Liu HH, Chen X, Seth RB, et al (2006) The specific and essential role of MAVS in antiviral innate immune responses. Immunity 24: 633–642. 16713980 10.1016/j.immuni.2006.04.004

[51] Zhong B, Yang Y, Li S, Wang YY, Li Y, et al (2008) The adaptor protein MITA links virus-sensing receptors to IRF3 transcription factor activation. Immunity 29: 538–550. 18818105 10.1016/j.immuni.2008.09.003

[52] Xu M, Skaug B, Zeng W, Chen ZJ (2009) A ubiquitin replacement strategy in human cells reveals distinct mechanisms of IKK activation by TNFalpha and IL-1beta. Mol Cell 36: 302–314. 19854138 10.1016/j.molcel.2009.10.002 PMC2779160

[53] Unterholzner L, Keating SE, Baran M, Horan KA, Jensen SB, et al (2010) IFI16 is an innate immune sensor for intracellular DNA. Nat Immunol 11: 997–1004. 20890285 10.1038/ni.1932 PMC3142795

