# Supplementary material for: COX5B Regulates MAVS-mediated Antiviral Signaling through Interaction with ATG5 and Repressing ROS Production
Source: PLoS Pathog. 2012 Dec 20;8(12):e1003086. doi: 10.1371/journal.ppat.1003086 (PMC3534373; doi:10.1371/journal.ppat.1003086)
Supplement: Text S1 — contains supplementary Figures S1 to S11 and one supplementary Table S1. (PDF) [file ppat.1003086.s001.pdf]

A

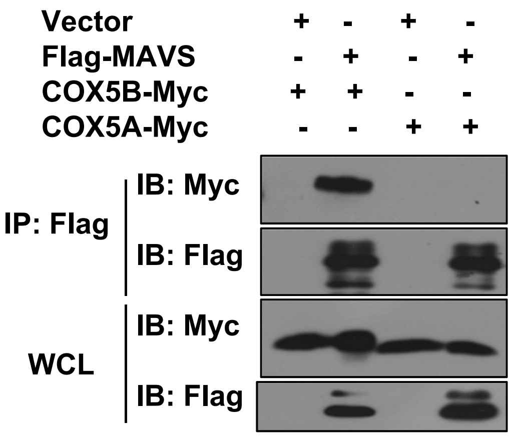

B

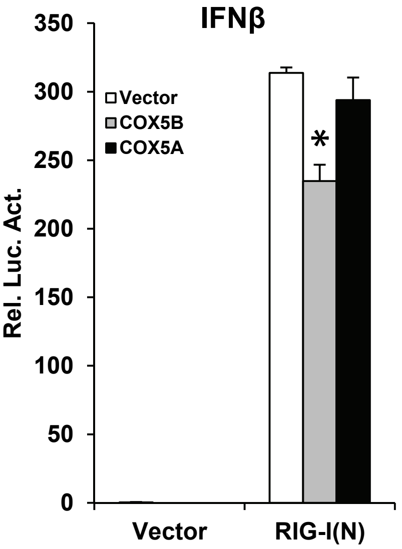

Figure S1 Overexpression COX5A fails to inhibit MAVS-mediated antiviral signaling
(A) HEK293 cells were transfected with the indicated plasmids, cell lysates were immunoprecipitated with anti-Flag beads, and then followed by immunoblot analysis.
(B) HEK293 cells were transfected with expression vectors for the indicated proteins together with IFNβ-luc and pRSV/LacZ vectors. Subsequently, cells were lysed for luciferase assays. Data from B are representative of at least three independent experiments (mean and s.d. of duplicate assays). *, $P < 0.05$; versus control groups.

A

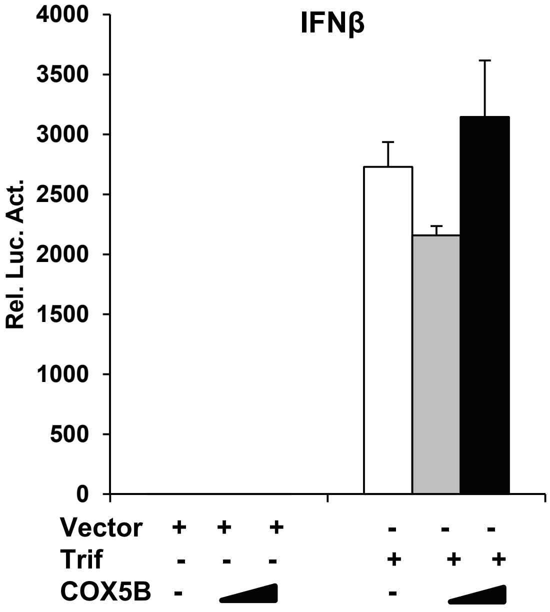

B

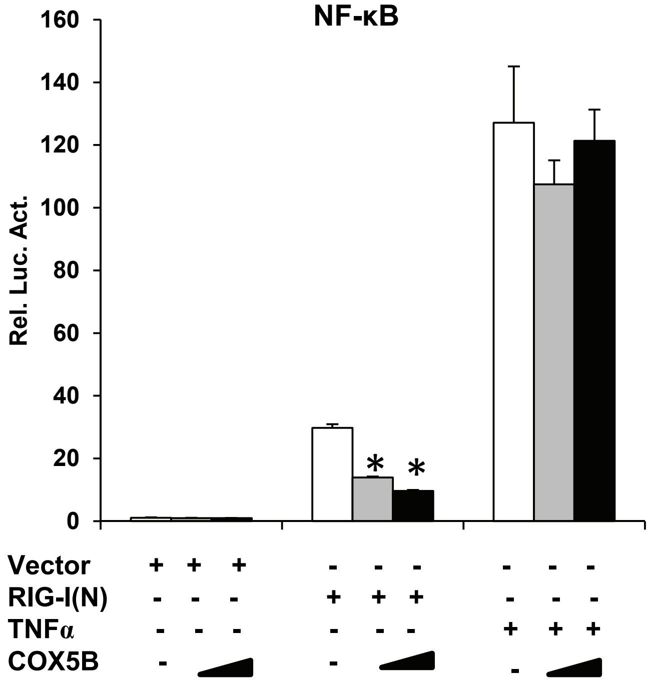

Figure S2 Overexpression COX5B suppresses the activation of IFN-β and NF-κB promoters specifically through MAVS signaling

(A) The Trif expressing vector (or empty vector) was co-transfected with increasing amount DNA of COX5B together with IFNβ-luc and pRSV/LacZ plasmids in HEK293 cells. After 24 h transfection, cells were lysed for luciferase assays.

(B) HEK293 cells were transfected with the indicated plasmids together with NF-κB-luc and pRSV/LacZ vectors. For TNFα treatment, after 24 h transfection, cells were treated with TNFα (20ng/ml) for another 7 h, and then lysed for luciferase assays.

Data from A and B are representative of at least three independent experiments (mean and s.d. of duplicate assays). *, $P < 0.05$ versus control groups.

A

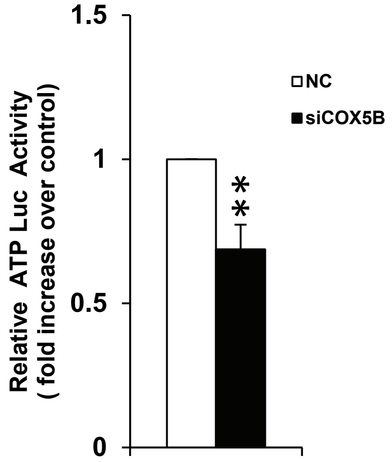

Figure S3 Knockdown of COX5B reduces ATP production

HEK293 cells were transfected with NC or COX5B RNAi oligos, then cells were lysed and the total cellular ATP was measured in a luminometer according to the protocol of ENLITEN ATP Assay System Bioluminescence Dection Kit (Promega). Data are presented as the mean ± SD from four independent experiments. **, $P < 0.01$ versus control (NC) groups.

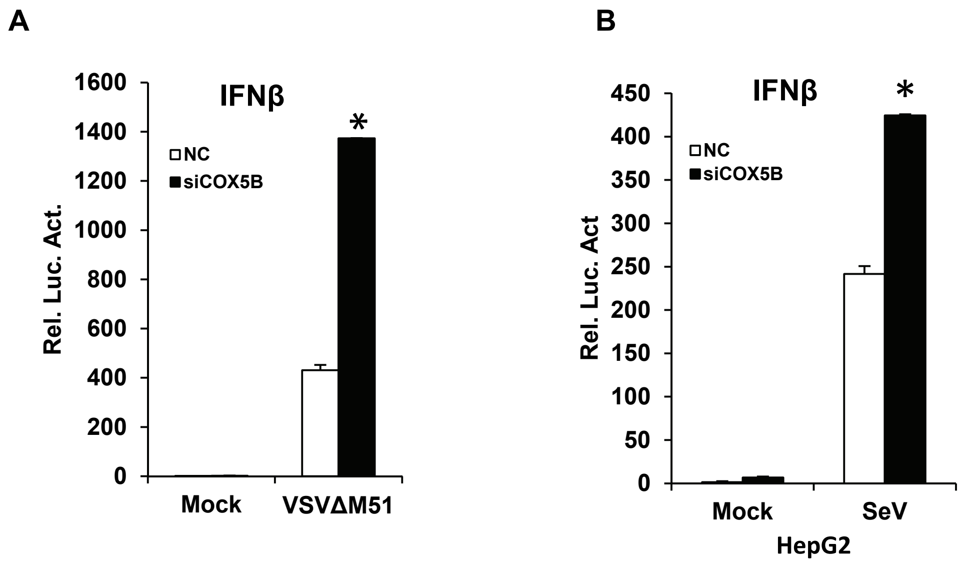

Figure S4 COX5B RNAi results in increased activation of IFNβ promoter

(A) HEK293 cells were transfected with NC or COX5B RNAi oligos, After 48 h transfection, IFNβ-luc and pRSV/LacZ plasmids were transfected into the knockdown cells, followed by the VSVΔM51-GFP (MOI=0.1) infection for 10 h, and subsequently cells were lysed for luciferase assays.

(B) HepG2 cells were first transfected with NC or COX5B RNAi oligos. After 48 h transfection, IFNβ-luc and pRL-TK renilla luciferase plasmids were transfected into the RNAi cells, followed by the Sendai virus (50HA unit /ml) infection for 16 h, and subsequently cells were lysed for luciferase assays, and normalized with renilla luciferase activity. Data from A and B are representative of three independent experiments (mean and s.d. of duplicate assays). *, $P < 0.05$ versus control groups.

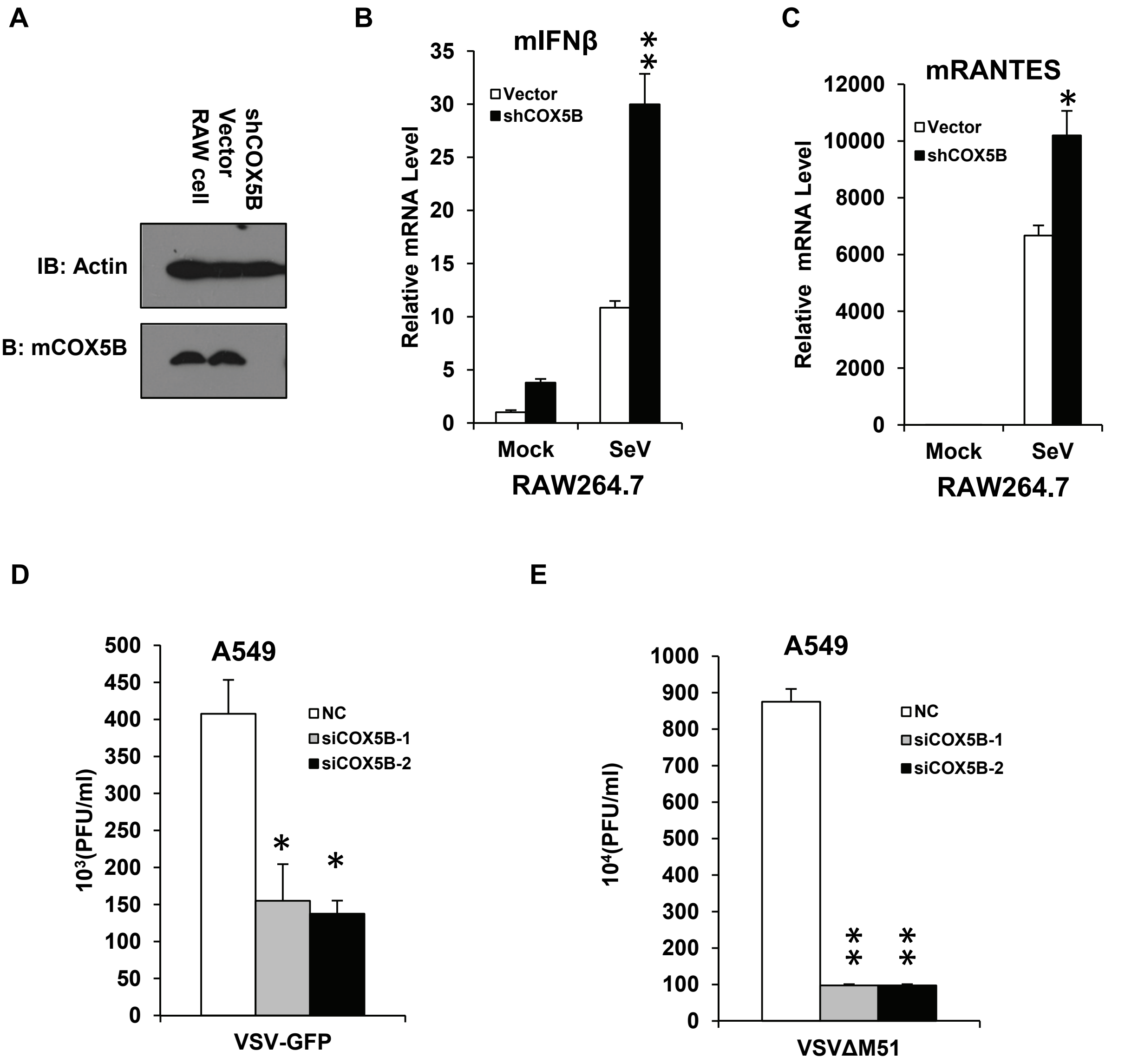

Figure S5 Knockdown of COX5B potentiates the antiviral responses and mediates the replication of virus

(A-C) RAW264.7 cells were transfected with pSUPERIOR-puro-vector and pSUPERIOR-puro-4*sh-mouse-COX5B using lipofectamine 2000 (Invitrogen), 20 h after transfection, then selected in puromycin (AMRESCO) at the concentration of 2μg/ml for one week. The shRNA stable clones which show efficient knockdown of endogenous mouse COX5B were used for experiments. The efficiency of COX5B knockdown in COX5B shRNA stable cells was confirmed by immunoblotting with a mouse COX5B antibody (A). (B-C) Control and mouse COX5B shRNA stable cells were infected with SeV (50HA unit/ml) for 18 h, then RNA was extracted for real-time PCR analysis.

(D-E) A549 cells were transfected with COX5B RNAi or control oligos, after 48 h transfection, cells were infected by VSV-GFP (D) or VSVΔM51-GFP (E) at the MOI of 0.01 for 12 h, subsequently the culture supernatant were collected for viral titer assays. Data from B-E are representative of three independent experiments (mean and s.d. of duplicate or triplicate assays). *, $P < 0.05$; **, $P < 0.01$ versus control groups.

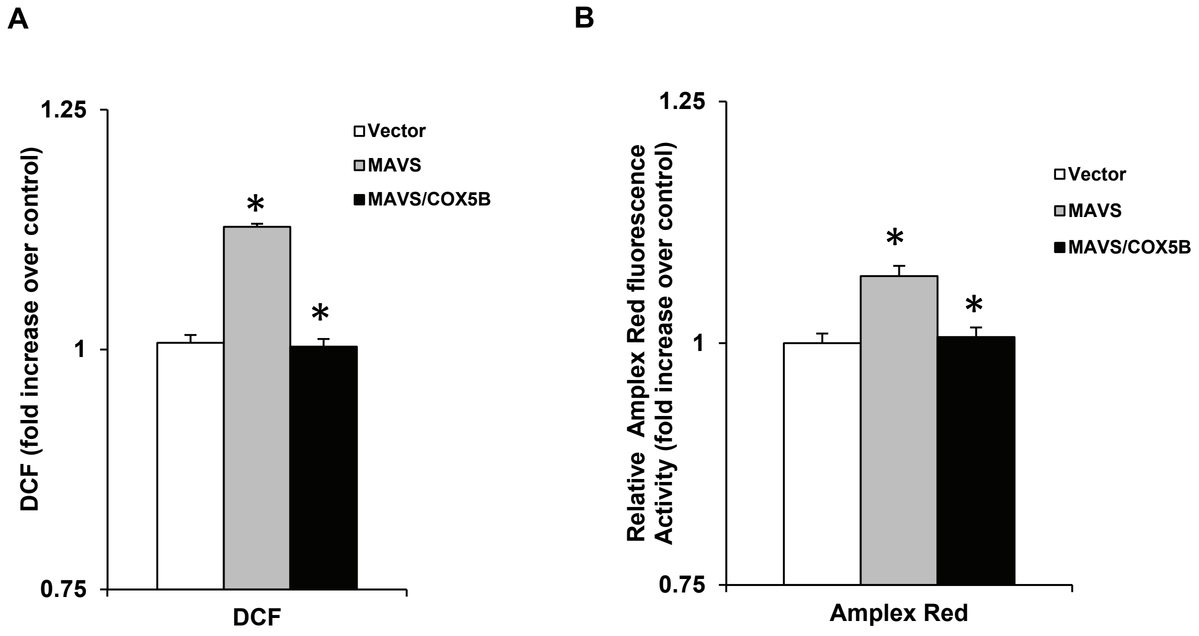

Figure S6 Overexpression of MAVS enhances ROS production

(A) HEK293 cells were transfected with the indicated plasmids for 30 h, and then stained with DCF, followed by FACS analysis.

(B) HEK293 cells were transfected with the indicated plasmids for 30 h, and then stained with Amplex Red for 30 mins to measure the fluorescence activity in a luminometer. The fluorescence was measured using excitation in the range of 530 to 560 nm and emission detection at approximately 590 nm.

Data from A and B are presented as the mean ± SD from at least four independent experiments. *, $P < 0.05$ versus control groups.

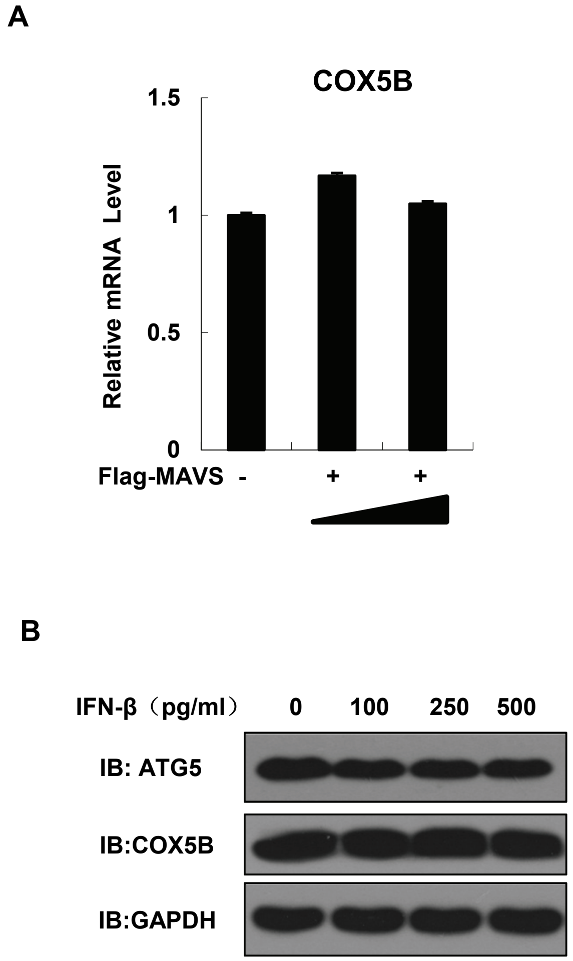

Figure S7 Overexpression of MAVS or IFNβ treatment affects COX5B and ATG5 expression

(A) HEK293 cells were transfected with increasing amounts of MAVS expression plasmids, and empty vector was used to balance the total DNA amount. Total RNA was extracted for real-time PCR analysis at 24 h post-transfection.

(B) HEK293 cells were treated with increased dosage of human IFNβ proteins (Sino Biological Inc.) for 12 h, and the total proteins were then lysed and subjected to immunoblot analysis.

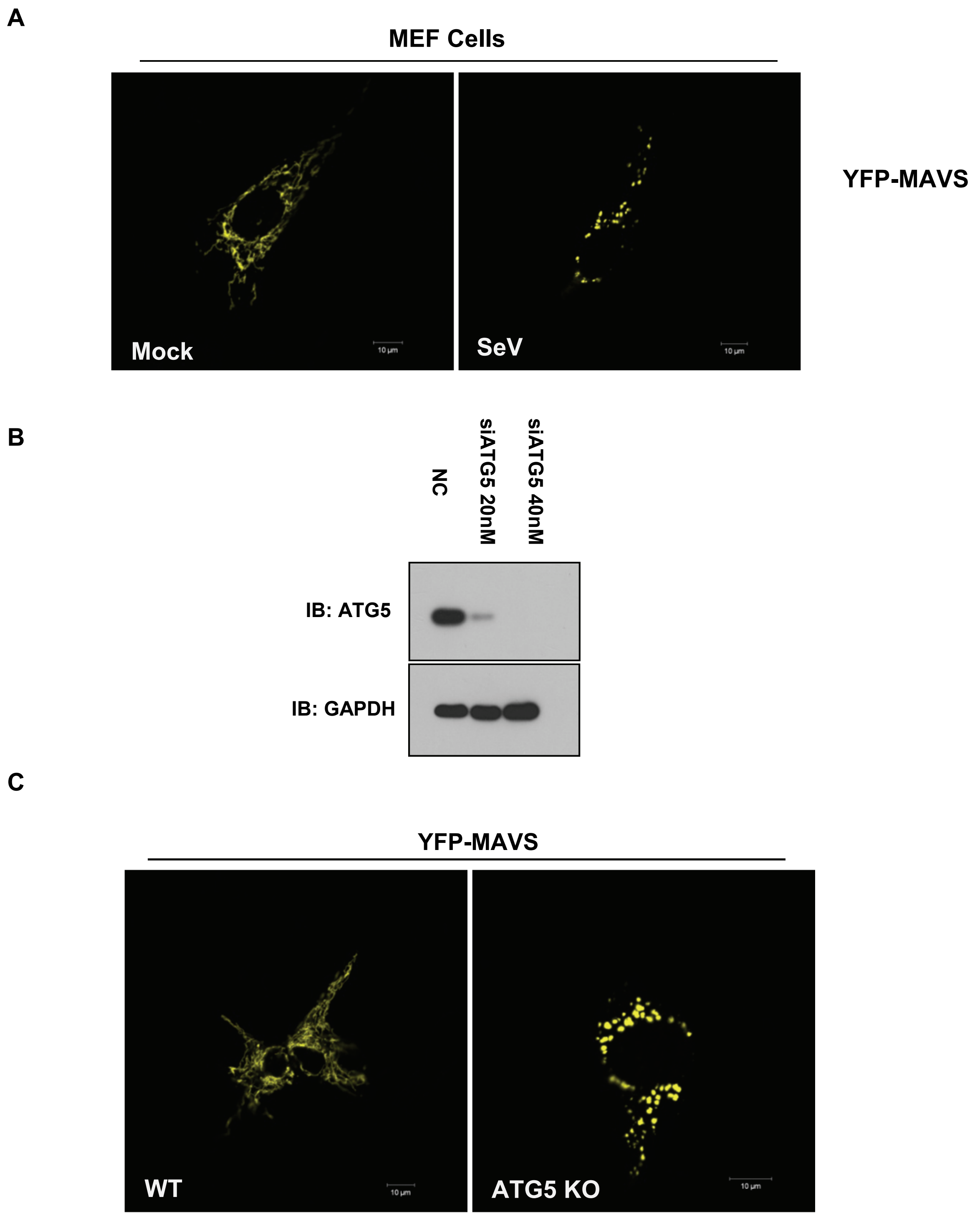

Figure S8 ATG5 knockdown effectively increases MAVS aggregation

(A) MEF cells were transfected with YFP-MAVS for 20 h, then infected with Sendai virus (50 HA unit/ml) for 16 h, and imaged by confocal microscopy.

(B) HEK293 cells were transfected with NC or ATG5 RNAi oligos. Thirty-six hours after transfection, cell lysates were analyzed by immunoblotting with the ATG5 antibody.

(C) WT and ATG5 KO MEF cells were transfected with YFP-MAVS for 30 h, then mounted onto slides directly, and imaged by confocal microscopy.

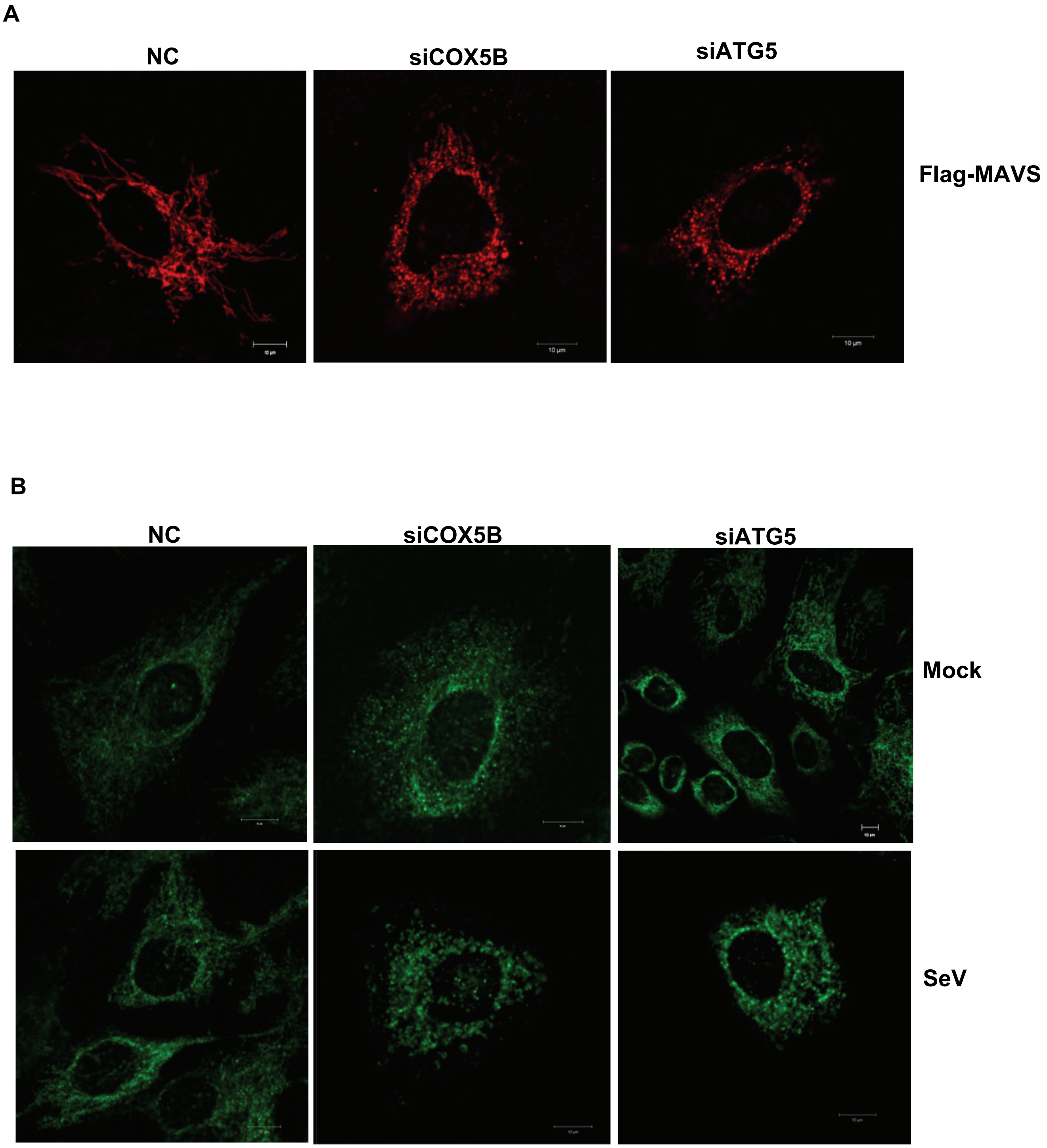

Figure S9 Knockdown of ATG5 or COX5B increases MAVS aggregation

(A) Hela cells were transfected with RNAi oligos of COX5B, ATG5 or NC, 24 h after oligo transfection, the cells were then transfected with Flag-MAVS, 30 h after plasmid transfection, cells were used to be stained with anti-Flag antibody for immunostaining analysis.

(B) Hela cells were transfected with RNAi oligos of COX5B, ATG5 or NC, 36 h after oligo transfection, the cells were then infected with Sendai virus (50HA uint/ml) for 20 h, then stained with anti-MAVS antibody, and imaged by confocal microscopy.

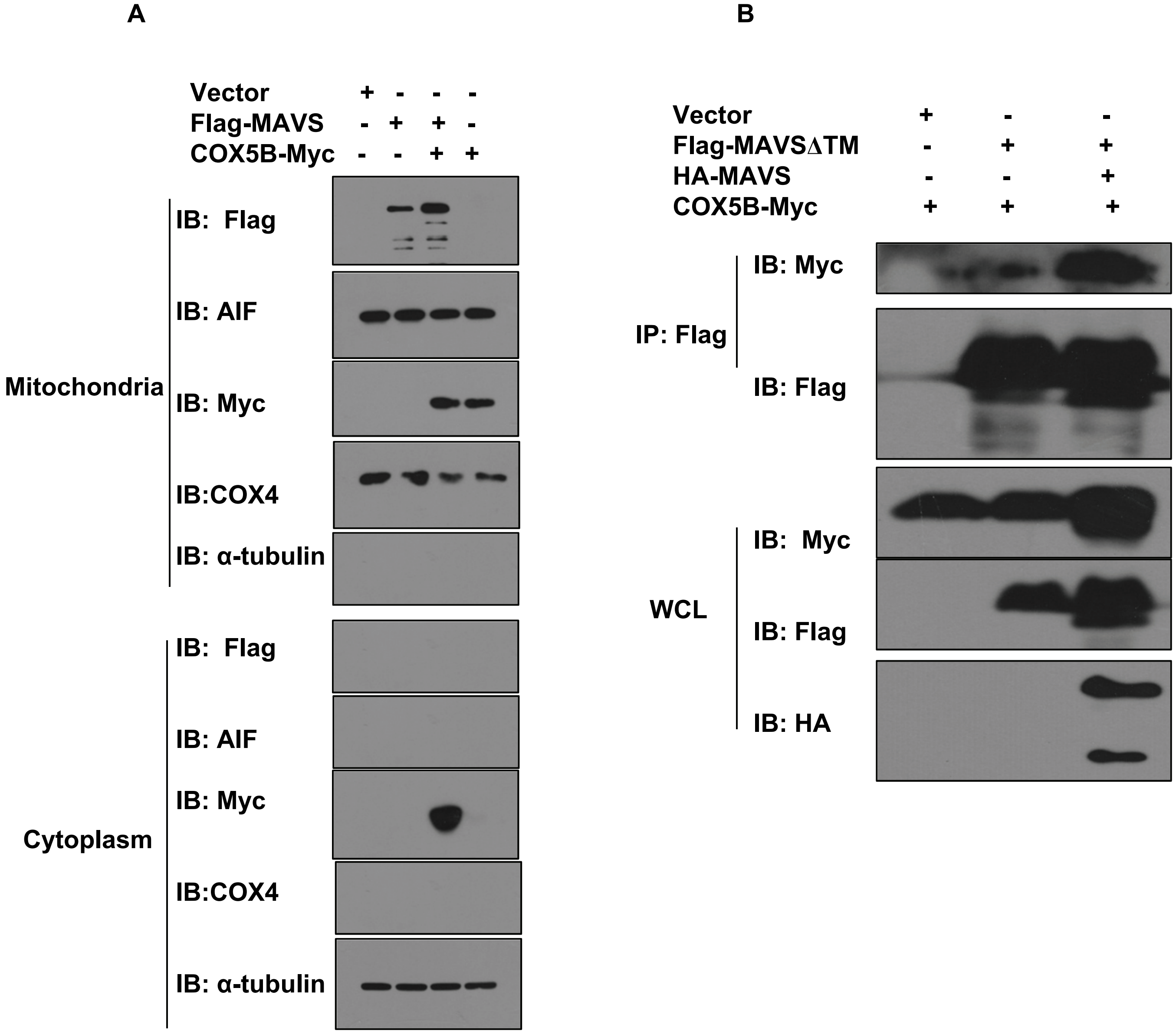

Figure S10 Overexpression MAVS increases levels of COX5B in cytosol

(A) HEK293 cells were co-transfected with Flag-tagged MAVS together with Myc-tagged COX5B or control vector. Twenty-four hours after transfection, cells were lysed to separate mitochondrial P5 and cytosolic fraction (S5). The separated fractions of mitochondrial P5 and cytosol S5 were then subjected to immunoblot analysis by indicated antibodies.

(B) HEK293 cells were transfected with Flag-tagged MAVSΔTM and Myc-tagged COX5B together with HA-tagged MAVS or control vectors. Twenty-four hours after transfection, cell lysates were immunoprecipitated with anti-Flag beads, followed by immunoblot analysis with the indicated antibodies.

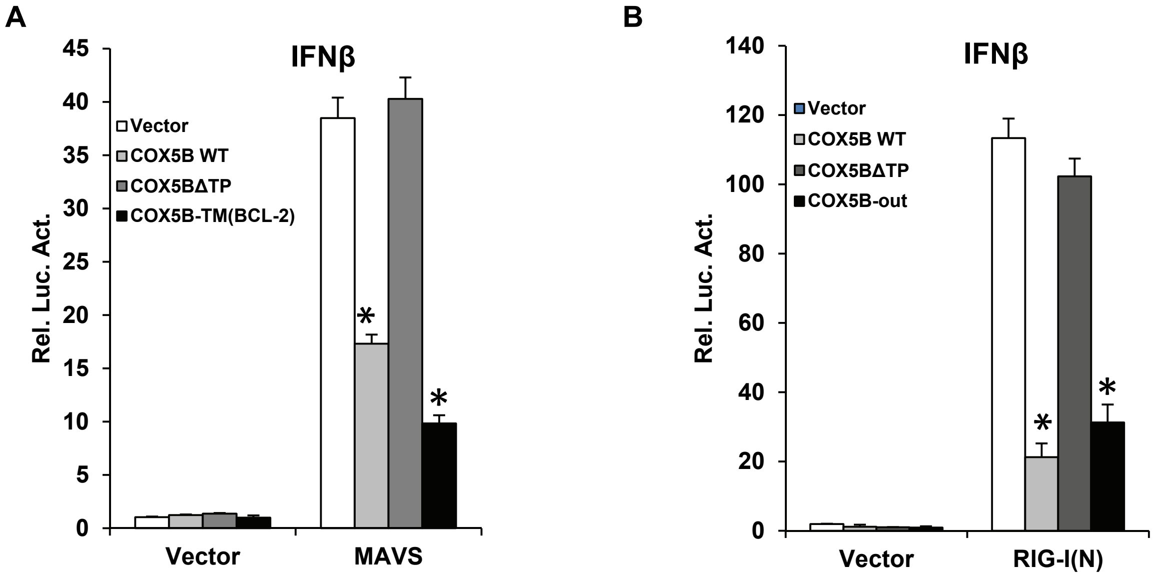

Figure S11 Expression of a mutant form of COX5B targeting to mitochondrial outer membrane suppresses MAVS signaling

(A and B) HEK293 cells were transfected with the indicated constructs together with IFNβ reporter plasmids. Thirty hours after transfection, cells were lysed for luciferase assays. Data are representative of three independent experiments (mean and s.d. of duplicate assays). *, $P < 0.05$; versus control groups.

Table S1. List of other MAVS-CARD interacted proteins, which were identified by yeast two-hybrid screening

Protein Name	NCBI Number
TPT1	NM_003295.2
AHSG	NM_001622.2
CFL1	NM_005507.2
SUOX	NM_000456.2
CDC42BPB	NM_006035.3
IFI6	NM_002038.3
HMGCS1	NM_001098272.2
MTHFS	NM_001199758.1
SERPING1	NM_000062.2
